# Osteoarthritis in the Elderly Population: Preclinical Evidence of Nutrigenomic Activities of Flavonoids

**DOI:** 10.3390/nu16010112

**Published:** 2023-12-28

**Authors:** Flores Naselli, Daniele Bellavia, Viviana Costa, Angela De Luca, Lavinia Raimondi, Gianluca Giavaresi, Fabio Caradonna

**Affiliations:** 1 Department of Biological, Chemical and Pharmaceutical Sciences and Technologies (STEBICEF), Section of Cellular Biology, University of Palermo, 90133 Palermo, Italy; flores.naselli@unipa.it (F.N.); fabio.caradonna@unipa.it (F.C.); 2 IRCCS Istituto Ortopedico Rizzoli, SC Scienze e Tecnologie Chirurgiche—SS Piattaforma Scienze Omiche per Ortopedia Personalizzata, 40136 Bologna, Italyangela.deluca@ior.it (A.D.L.); lavinia.raimondi@ior.it (L.R.); gianluca.giavaresi@ior.it (G.G.); 3 NBFC, National Biodiversity Future Center, 90133 Palermo, Italy

**Keywords:** osteoarthritis, inflammation, oxidative stress, flavonoids, nutrigenomics

## Abstract

Osteoarthritis (OA) is a degenerative joint disease that is age-related and progressive. It causes the destruction of articular cartilage and underlying bone, often aggravated by inflammatory processes and oxidative stresses. This pathology impairs the quality of life of the elderly, causing pain, reduced mobility, and functional disabilities, especially in obese patients. Phytochemicals with anti-inflammatory and antioxidant activities may be used for long-term treatment of OA, either in combination with current anti-inflammatories and painkillers, or as an alternative to other products such as glucosamine and chondroitin, which improve cartilage structure and elasticity. The current systematic review provides a comprehensive understanding of the use of flavonoids. It highlights chondrocyte, cartilage, and subchondral bone activities, with a particular focus on their nutrigenomic effects. The molecular mechanisms of these molecules demonstrate how they can be used for the prevention and treatment of OA in the elderly population. However, clinical trials are still needed for effective use in clinical practice.

## 1. Introduction

Osteoarthritis (OA) is usually a long-term degenerative condition linked to the aging process and obesity. It is characterized by the gradual breakdown of joint cartilage, which causes joint pain and impairs joint function [1]. Osteoarthritis (OA) can have a serious negative impact on an individual’s quality of life. It causes joint pain, reduced mobility, and functional and social impairments, which can lead to depression and impose a significant social burden. The incidence of osteoarthritis is influenced by many factors, such as work, sports participation, musculoskeletal injuries, obesity, and gender [2,3,4]. This burden is particularly noteworthy in the aging population of industrialized countries, due to the combination of increased life expectancy, the growing size of the elderly population, and the high incidence of osteoarthritis among both elderly and obese individuals.

Articular cartilage is a dense connective tissue without nerves, blood vessels, or lymphatic vessels; thus, it lacks an intrinsic repair system. Chondrocytes are the only cell type present in articular cartilage, and they secrete growth factors and enzymes to regulate the synthesis of the extracellular matrix (ECM), which predominantly contains collagen-II (Col-II) and aggrecan (ACAN), and maintain a delicate balance between synthesis and degradation of the ECM. The ECM network produced by chondrocytes plays a protective role, absorbing mechanical stress during joint movement [5]. However, any damage to the articular cartilage determines a low-grade inflammation, which favors the catabolism operated by enzymes such as matrix metalloproteinases (MMPs) and a disintegrin and metalloproteinase with thrombospondin motifs (ADAMTS); inflammation and oxidative stress (OS) are the two deleterious entities that supply the progression of OA. In this unfavorable environment, chondrocytes proliferate and expand, becoming hypertrophic, and, over time, proceed towards apoptosis, being replaced by osteoblasts and then by bone tissue. This process is called endochondral ossification and, although it is natural in growing bone, it increases the progression of osteoarthritis [6].

**Figure 1:**
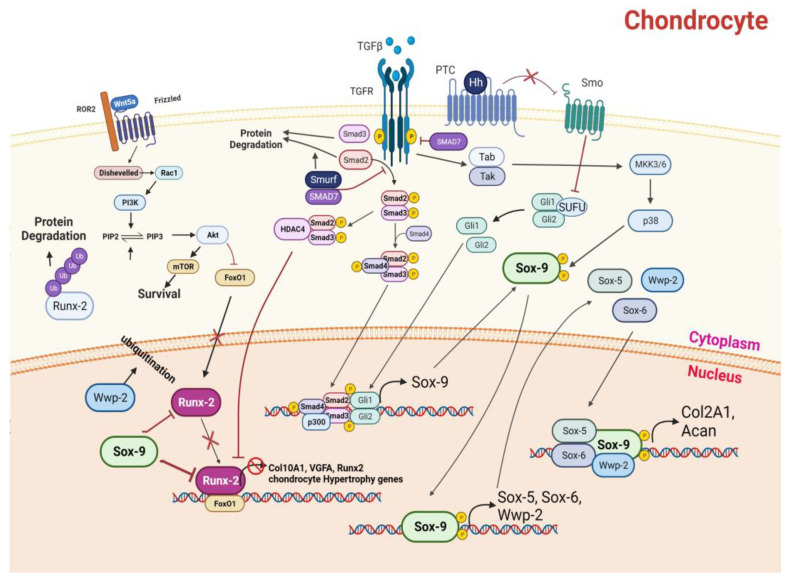
Schematic drawing representing the complexity of network pathways of chondrocyte differentiation (created with biorender.com).

Cartilage homeostasis is based on a dense network of pathways that can be perturbed by inflammation and/or oxidative stress, leading to the initiation or progression of osteoarthritis. Chondrocyte differentiation is regulated by several signaling pathways that constitute a dense network, where Tumor Growth Factor-β (TGF-β), Hedgehog (Hh), Notch, and non-canonical wingless-type Mouse Mammary Tumor Virus (MMTV) integration site family member (Wnt) pathways (Wnt-5a) cooperate for the activation of SRY-Box Transcription Factor 9 (SOX-9), a high-mobility group domain transcription factor and master regulator of chondrogenesis (see Figure 1). TGF-β interacts with its receptor, activating Smad-2/3 through Smad-4 recruitment. Smad-7 and SMURF normally inhibit receptor autophosphorylation and the translocation of the nucleus, through Smad-2/3 degradation [6]. Patched-1 (PTC-1) interacts normally with smoothened (SMO), maintaining its inactive state. With interaction with Hh, SMO is free and leads to the liberation of GLI-1 and GLI-2 proteins by SUFU, and consequently, induces their nuclear translocation. The Smad-2/3–Smad-4 complex and GLI proteins converge on the SOX-9 promotor and induce its expression and activation [7].

SOX-9 is a master transcript factor responsible for chondrocyte commitment and is also stabilized through the phosphorylation operated by p38 MAPK, always activated by TGF-β signaling, where TAK/TAB actions determine the activity of MAPKK, MKK3/6 [8]. SOX-9 is responsible for the transcription of SOX-5, SOX-6, and WWP2, which with SOX-9 determine the expression of chondrocyte effector genes, such as Col2A1 and ACAN, constituting the ECM. WWP-2 is also responsible for runt-related transcription factor 2 (RUNX-2) ubiquitination and degradation [9,10,11,12,13,14]. Activation of the non-canonical Wnt-5a pathway determines, through Disheveled (DVL) and Ras-related C3 botulinum toxin substrate (RAC) activities, phosphatidylinositol 3-kinase (PI3K) activation, which catalyzes the phosphorylation of phosphatidyl inositol diphosphate (PIP2) in phosphatidyl inositol triphosphate (PIP3), activating protein kinase B (AKT) and, consequently, mammalian target of rapamycin (mTOR), favoring chondrocyte survival. Furthermore, AKT activation is also responsible for forkhead box protein O-1 (FoxO1) inhibition, causing RUNX-2 activity downregulation [15].

**Figure 2:**
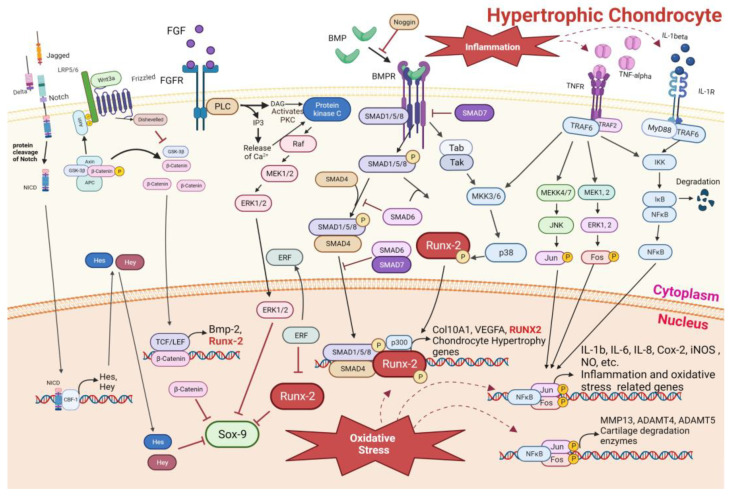
Schematic drawing representing altered network pathways during chondrocyte hypertrophy and the influence of inflammation and oxidative stress (created with biorender.com).

This system is maintained in equilibrium by SOX-9 activities and RUNX-2 downregulation. In fact, RUNX-2 is responsible for the maturation and hypertrophy of chondrocytes, which represent the terminal differentiation of chondrocytes that successively progress to apoptosis, and they are then substituted for by osteoblasts and bone. During osteochondral differentiation, the presence of osteoblast differentiation factor, Wnt3a, parathyroid hormone (PTH), Bone Morphogenic Proteins (BMPs), fibroblast growth factor (FGF), and Notch determine the inactivation of SOX-9 and activation of RUNX-2 factor, implicated in the hypertrophy of chondrocytes (see Figure 2), with the expression of Col10A1 and VEGFA [16]. In particular, canonical Wnt signaling protein (Wnt3a) interacts with Frizzled and Low-Density Lipoprotein 5/6 (LRP-5/6), determining Axin binding and the disruption of the Axin/Adenomatous Polyposis Coli (APC)/Glycogen Synthase Kinase-3β (GSK-3β) complex, which is responsible for β-catenin degradation. Canonical Wnt signaling is inhibited by Dikkopf (Dkk), kremen, sclerostin factors, and Secreted Frizzled-Related Protein (sFRP), blocking the first three LRP-5/6 cofactors, while the last binds Wnt3a molecules. β-catenin interacts with T-cell factor (TCF) and lymphoid enhancer-binding factor (LEF), as well as BMP-2 and RUNX-2 expression. PTH signaling increases β-catenin stabilization and activity [17]. BMPs bind their receptors (BMPRs), activating the small-worm phenotype (SMA)- and mothers against decapentaplegic (MAD)-Related Protein 1/5/8 (Smad-1/5/8)/Smad-4 complex, and consequently, the expression of RUNX-2 in the nucleus. The BMP pathway determines the kinase signaling cascade that ends with p38-kinase activation, a mitogen-activated protein kinases (MAPKs) family member, which leads to the activation by phosphorylation of RUNX-2. Noggin inhibits BMP pathway-binding BMP molecules, and Smad-6 and Smad-7 induce Smad-1/5/8 degradation, preventing Smad-1/5/8 complex activation, inhibiting BMPR auto-phosphorylation, and inhibiting nuclear translocation [18]. Proteolytic cleavage of Notch receptors after the interaction of their ligands (Delta and Jagged) determines the Notch intracellular domain (NICD) release and its nuclear translocation, where, with Core-Binding Factor-1 (CBF-1), it determines the transcription of HES and HEY, which negatively regulate SOX-9 [14]. FGF pathway activation determines phospholipase C (PLC) activities, catalyzing the scission of PIP3 in inositol triphosphate (IP3) and diacylglycerol (DAG). DAG formation induces protein kinase C (PKC) activity that increases MAPK activation. This activates extracellular signal-regulated kinase (ERK) proteins determining both SOX-9 inactivation and nuclear exportation of ERF protein, an inhibitor of RUNX-2, improving RUNX-2 dependent hypertrophy [19].

Inflammation, whose main mediators are IL-1β and TNF-α, determines the activation of two signaling pathways that converge with different cofactors, TNF-α with TRAF2 and IL-1β with MyD88, in TRAF-6 factor. TRAF-6 mediates the activation of all three types of MAPKs (ERK, JNK, and p38MAPK) and NF-kB. These signals determine the expression of cartilage degradation enzymes as well as the genes implicated in inflammation and oxidative stress, such as IL-1β, IL-6, IL-8, COX-2, etc. [20]. It is known that the oxidative stresses caused by ROS, such as hydrogen peroxide (H_2_O_2_) and peroxynitrite, are capable of triggering and accelerating the terminal differentiation processes of chondrocytes, which become hypertrophic and produce a secretome similar to that of senescent cells. In addition to producing type X collagen, chondrocytes also begin an inflammatory secretome process typical of senescent cells (senescence-associated secretory phenotype, SASP) which ultimately leads to programmed cell death, establishing and aggravating osteoarthritis [21]. Furthermore, it has been observed that oxidative stresses determine the reduction in Smad-2 and Smad-3 levels in hypertrophic chondrocytes. A reduction in Smad-2 results in the activation of necrotic cell death pathways, as indicated by specific inhibitors of Smad-2, without the induction of apoptosis. Meanwhile, the reduction in Smad-3 exclusively determines an increase in apoptosis (both early and late) as indicated by the use of the Smad-3 inhibitors [22].

**Figure 3:**
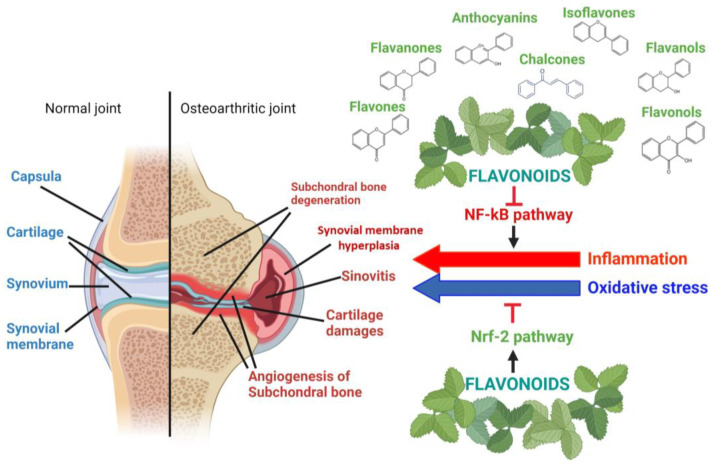
Schematic drawing of the difference between a normal and osteoarthritic joint and activities of all classes of flavonoids (anthocyanins, chalcones, flavanols, flavanones, flavones, flavonols, and isoflavones) on an OA joint through specific pathways (created with biorender.com).

Medical herbs have been used since antiquity to treat several pathologies due to their antioxidant and anti-inflammatory activities. Recently, various phytochemicals, known for their potent anti-inflammatory and antioxidant activities, have been studied for their potential use in treating degenerative musculoskeletal pathologies, such as osteoporosis [23,24,25,26,27]. Most of these molecules are flavonoids and could be suitable for long-term use in OA, in combination with both anti-inflammatories and pain relievers already in clinical use, or as an alternative to other products such as glucosamine and chondroitin, which are believed to improve the structure and elasticity of cartilage [27]. Flavonoids occur naturally in all plant parts, such as fruits, leaves, flowers, tree bark, and roots, and act by activating or inhibiting specific pathways (see Figure 3) [28]. In this review, we provide an exhaustive literature overview of in vitro, in vivo, and in silico studies on flavonoids isolated from plants in the past five years, to evaluate their effects on articular cartilage and their potential nutrigenomic effects [29], suggesting that they could be used to treat OA, acting on inflammation and oxidative stress.

## 2. Research Strategies and Results of Collected References on Flavonoids and Osteoarthritis

**Figure 4:**
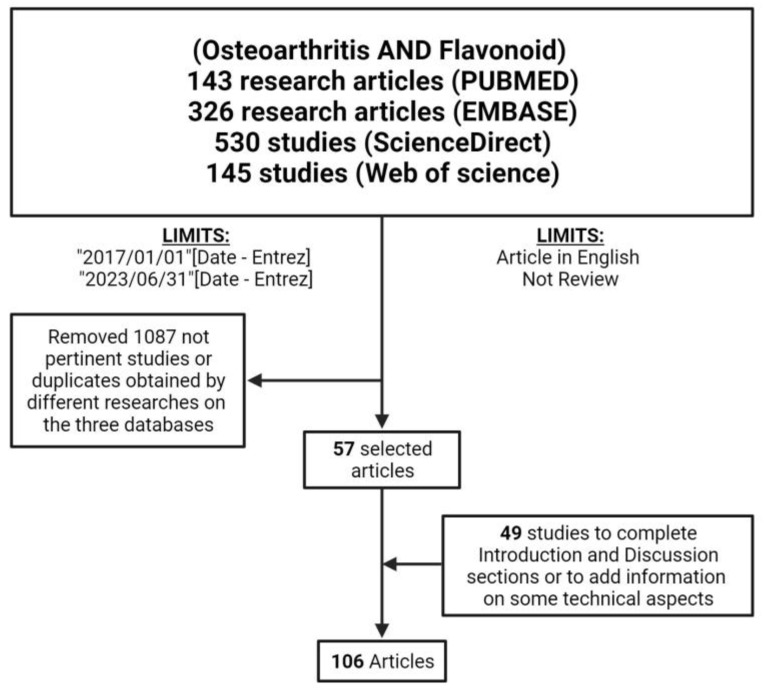
Flowchart of the research strategy and selection of bibliographic references (created with biorender.com).

For this study, we conducted literature research in four different databases: specifically, the databases searched were MEDLINE, ScienceDirect, EMBASE, and the Web of Science. The search query employed for bibliographic research was “Osteoarthritis AND Flavonoid”. A search of the MEDLINE database using the PubMed research engine conducted in the English language (AND (English(Filter))) and published in the last five years (AND (y_5(Filter))) retrieved 153 articles. Of these, reviews (NOT Review (Publication Type)) were excluded, reducing the number of collected articles to 143. A search was conducted using the same strings and limitations (year range, language, and research article) in ScienceDirect, EMBASE, and the Web of Science databases, which found 530, 326, and 145 articles, respectively. Five reviewers (F.N., D.B., V.C., L.R. and A.D.L.) manually assessed the titles and abstracts of the collected references to discard duplicates or non-pertinent articles (e.g., research articles that did not use isolated compounds). Then, 57 articles related to the topics of the review were selected. An additional 49 references were cited to provide detailed technical aspects for a better understanding of the mechanisms involved in cartilage regulation and OA development. A detailed flowchart of research strategies is presented in Figure 4.

Of the 57 articles selected, 17 contained in vitro studies, 33 contained in vitro and in vivo studies, and 9 contained only in vivo studies; no clinical studies were found. Regarding in vitro studies, the molecules used to create an inflammatory condition resembling OA in chondrocytes were:

- Interleukin (IL)-1β (64%, 32/50);
- Lipopolysaccharides—LPSs (8%, 4/50);
- Tumor necrosis factor-α—TNF-α (4%, 2/50);
- H_2_O_2_ (2%, 1/50);
- Advanced glycation end products—AGE (2%, 1/50);
- Sodium nitroprusside—SNP (2%, 1/50);
- Angiotensin II—ANG-II (2%, 1/50).

**Figure 5:**
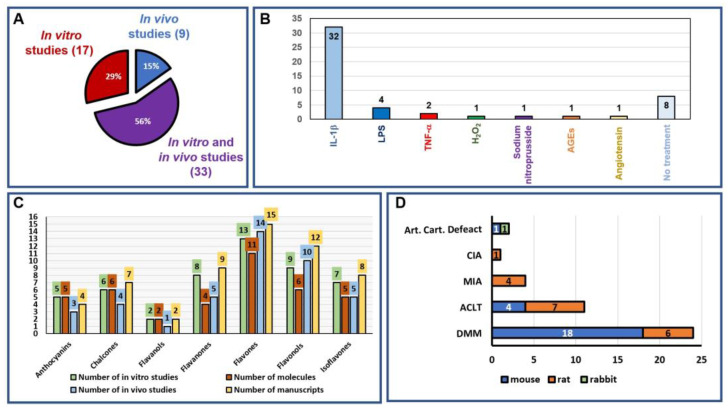
Graphical results of bibliographic research. (**A**) Among 45 selected manuscripts, 32% are studies carried out using only in vitro models, 8% only in vivo, and 60% investigated flavonoid activities in both in vitro and in vivo models. (**B**) Modalities of OA induction in in vitro studies. (**C**) Number of in vitro studies, in vivo studies, studied molecules, and selected manuscripts, using the different classes of flavonoids. (**D**) Modalities of OA induction in in vivo models, distinguishing between rat, mouse, and rabbit (created with biorender.com).

Within the height studies, which accounted for 16% of the total, the chondrocytes were isolated either from animals or patients who had osteoarthritis (OA) or were otherwise normal. Of these in vitro studies, 34% (17/50) only reported in vitro experiments, while the remaining 66% presented both in vitro and in vivo studies. For in vivo studies, 14.3% (9/42) reported exclusively in vivo experiments, while the remaining 78.6% (33/42) used both in vivo and in vitro experiments to identify the pathways involved. All in vivo studies used small-animal models (54.8% mice, 42.9% rats, and 2.4% rabbits), in which different techniques to induce OA were used. The most frequently used technique to induce OA was surgical destabilization of the medial meniscus (DMM, 24/42, 57.1%); the other models to induce OA were anterior cruciate ligament transection (ACLT, 11/42, 26.2%), mono-iodoacetic injection (MIA, 4/42, 9.5%), collagen-induced arthritis (CIA, 1/42, 2.4%), and articular cartilage defect (2/42, 4.8%). All these analyses are summarized in Figure 5.

All the evidence emerging from the collected studies on the ability of flavonoids to improve cartilage homeostasis and limit OA progression are reported in the following paragraphs.

## 3. Flavonoid Classes and Activities on OA

Flavonoids are secondary metabolites produced by plants and have an integral role in their growth and development. They consist of a broad range of common plant compounds with a flavone ring as the backbone structure. These compounds have been found to have a beneficial effect through a wide range of pathologies such as neurodegenerative diseases, diabetes, tumors, and osteoporosis. Several studies have already shown the potential OA-specific effects of flavonoids; these results are reported separately for each flavonoid subfamily: **anthocyanins, chalcones, flavanols, flavanones, flavones, flavonols, and isoflavones.** In particular, we describe the nutrigenomic effects of these classes of molecules, focusing on the pathway of interest for these actions for both “in vitro” and “in vivo” studies. Table 1 lists all the selected articles in which flavonoids were involved, emphasizing their roles in the regulation of chondrocyte metabolism (Table 1).

**Anthocyanins** are a subclass of the flavonoid family that are synthesized through the phenylpropanoid pathway and provide a variety of health-promoting benefits due to their antioxidative properties. Four articles focused on five anthocyanin molecules: cyanidin (cyanidin-3-glucoside chloride, C3G), delphinidin, malvidin, pelargonidin (PG) and procyanidin B2 (PCB2) [30,31,32,33]. C3G is a flavonoid found in grape seeds and blueberries that has several pharmacological properties, including anti-inflammatory and antioxidative activities, as well as anti-apoptotic and anti-tumoral effects. Saulite et al. (2019) demonstrated that different anthocyanins, such as C3G, delphinidin, and malvidin, can influence adipocyte-derived mesenchymal stem cell differentiation. C3G and delphinidin promote chondrogenesis, as indicated by the expression of articular chondrocyte markers such as Col-II and ACAN, and by staining with Alcian Blue of spheroids. Delphinidin is also an inhibitor of adipogenesis, resulting in decreased expression of fatty acid-binding protein-4 (FABP-4), lipoprotein lipase (LPL), and adiponectin, as well as reduced Oil Red O staining. Malvidin seems to have no effect on chondrogenic differentiation, but significantly improves OB differentiation, with possible involvement in the subchondral bone microenvironment [30]. Wang et al. (2019) carried out an in vivo study indicating how C3G tail vein injection significantly alleviated bovine type II collagen-induced arthritis (CIA) in Sprague Dawley rats. C3G increased the proportion of the regulatory T (T-reg) cell lymphocyte subpopulation and decreased CD38+ NK cells. Concurrently, they observed an increase in released interleukin (IL)-10, with potent anti-inflammatory actions, and the IL-6 and interferon (IFN)-γ levels in synovial fluid. C3G exerted its action by increasing Sirtuin-6 (Sirt-6) expression, an NAD+-dependent deacetylase, as demonstrated using Sirt-6 siRNA, which reversed the effect of C3G treatment. In fact, CIA rats treated with both C3G and Sirt-6 inhibitor OSS_128167 showed joint inflammation and a low Treg cell lymphocyte subpopulation, although the CD38+ NK subpopulation results were still low [31]. PG, an anthocyanin abundant in berries with a demonstrated redox and anti-inflammatory power, is able to positively influence cartilage deterioration in OA by modulating inflammation response, as reported by Zeng et al. (2023). In this study, the in vivo approach was utilized as a basis to determine, in vitro, the right PG concentration, to demonstrate that PG (i) improves inflammatory response, (ii) downregulates the typical inflammation markers, (iii) improves cartilage degeneration in osteoarthritis via suppressing the NF-κB pathway [32]. Cai et al. (2022) explored how PCB2 was able to reduce chondrocyte apoptosis and senescence, indicated as principal processes implicated in cartilage destruction and loss of ECM deposition. Senescent cells are tightly related to P-16^INK4A^ and P-21 expression, which leads to senescence-associated secretory phenotype (SASP), producing several inflammation-promoting factors (inflammatory cytokines and ECM proteases) and, thus, aggravating articular cartilage degeneration. PCB2 acted on chondrocytes, determining activation and nuclear translocation of the nuclear factor erythroid 2-related factor-2 (Nrf-2) and Heme Oxygenase-1 (HO-1) proteins. Nrf-2 inhibited the nuclear factor kappa-light-chain-enhancer of activated B cells (NF-κB) pathway, leading to an increase in chondrocyte viability and suppression of SASP [33].

**Chalcones** belong to a minor class of flavonoids characterized by a 1,3-diphenyl-2E-propene-1-one skeleton. They have several activities, such as anti-inflammatory, antioxidative, and anticancer. Seven manuscripts were collected regarding six chalcone molecules: butein, cardamonin, isoliquiritigenin (ISL), licochalcone A (Lico A), safflower yellow (SY), and xanthohumol (XN) [34,35,36,37,38,39,40]. Butein, 2′,3,4,4′-Tetrahydroxychalcone, was found to be a polyphenol produced by several plants, discovered in *Butea monosperma*. Ansari et al. (2018) showed how chondrocytes treated with IL-1β increased pro-inflammatory cytokine IL-6 release and demonstrated that butein administration was able to considerably reduce IL-6 production, as well as increase chondrocyte viability. In this work, the researchers indicated how these activities of butein are mediated by the induction of autophagy in human OA chondrocytes, through the activation of the mTOR pathway, which suppresses the expression of inflammatory mediators, and then the progression of OA pathology [34]. Cardamonin is a chalcone discovered in *Alpinia katsumadai*. This molecule was observed to be able to reverse the IL-1β-induced apoptosis of chondrocyte cell line CHON-001, as well as inhibiting pro-inflammatory cytokines expressions (IL-6, IL-8, and TNF-α). Cardamonin was also found to determine a reduction in enzymes implicated in ECM degradation, as well as inducing ECM expression. Jiang et al. (2021) showed how these actions are due to Nrf-2 activation, confirmed using si-RNA for Nrf-2, which can partially reverse these beneficial effects of cardamonin treatment on inflammation status and the ECM [35]. ISL is a trans-chalcone found in licorice. Ye et al. (2020) showed how ISL promotes the reduction in inflammation and fibrosis induced by ANG-II treatment, reducing ANG-II-induced activation and nuclear localization, and reversing the decrease in expression of PPAR-γ [36]. Ji et al. (2018) have evaluated the effects of ISL on the progression of osteoarthritis, using an ACLT mouse model. The results showed that ISL treatment reduced OA progression significantly and reduced the Osteoarthritis Research Society International (OARSI) score, protecting cartilage from destruction. ISL treatment increased lubricin expression while reducing the reduction in Col-X and MMP-13. Furthermore, the treatment reduced the aberrant subchondral bone modification, inhibiting osteoclastogenesis directly through the inhibition of the receptor activator of NF-κB (RANK)/RANK ligand (RANKL) signaling, and preventing abnormal bone formation by reducing transforming growth factor-β expression and release. Altogether, these results indicated that ISL attenuates OA progression by acting on alterations of both the cartilage tissue and subchondral bone [37]. Lico A is a chalcone compound extracted from the roots of the Glycyrrhiza species. Administration of Lico A showed protective effects on chondrocytes, inhibiting LPS-dependent activation of pyroptosis. This process is a lytic and inflammatory type of programmed cell death that is induced by inflammasomes and carried out by gasdermin proteins. Lico A inhibits inflammasome complex formation through Nrf-2/HO-1 expression and consequent NF-κB inhibition. An in vivo experiment on Lico A administration showed an attenuation of OA features in a DMM mouse model and reduced the OARSI score, indicating Lico A as a potential therapeutic molecule in OA [38]. SY is the main active component isolated from *Carthamus tinctorius*. In vitro experiments describe how SY treatments can revert the TNF-α dependent inflammatory response, inhibiting IL-1β, prostaglandin-endoperoxide synthase-2 (PTGS-2), and MMP-13, as well as inducing expression of the ECM (Col-II and ACAN). SY significantly reduces NF-κB activation and oxidative stress, by promoting AMP-activated protein kinase (AMPK) phosphorylation, along with Sirtuin-1 (Sirt-1), Nrf-2, and HO-1 expressions. To demonstrate these activities, the use of si-RNA targeting Nrf-2, blocking antioxidative actions, partially inhibited the benefit of SY treatment. In vivo experiments on SY administration in the ACLT rat model showed that the OA group, with respect to the sham-operated control group, presented an irregular profile of articular cartilage with reduced thickness. In the SY-treated OA group, cartilage degeneration was significantly reduced, with an increased thickness of cartilage [39]. Chen et al. (2021b) investigated the activities of XN, a prenylated flavonoid isolated from *Humulus lupulus* L., on OA. By treating OA chondrocytes with this molecule, mimicked through IL-1β treatment, they were able to significantly reduce pro-inflammatory cytokine expression, as well as ECM-degrading enzymes (MMP-13 and ADAMTS-5). XN administration in a DMM mouse model exhibited a reversion of the OA phenotype, indicated by the OARSI score. XN administration significantly slowed down the degenerative process in OA mice, also showing the nuclear translocation of Nrf-2, which essentially confirmed the central role of this factor as evidenced in in vitro experiments [40].

**Flavanols**, often called catechins, are found in a wide variety of fruits and vegetables. They have a 3-hydroxyflavone backbone, and they offer several benefits for various pathologies, particularly those with an inflammatory etiology. Two molecules, Epigallocatechin-3-O-gallate (EGCG) and Theaflavin-3,3’-Digallate (TFDG), have been studied in two manuscripts [41,42]. In Yang et al. (2022a), it was found that EGCG could alleviate chondrocyte injury, as mimicked in vitro by treating the CHON-001 cell line with IL-1β, reducing MMP-13 and IL-6 expression and release. EGCG’s activities appear to be associated with decreased levels of the microRNA miR-29b-3p, which targets phosphatase and tensin homolog (PTEN) mRNA, and then increases its expression, as indicated by the reversal of EGCG activities by MiR-29b-3p mimic. Further confirmation was provided by PTEN overexpression, which showed that it could abrogate the effects of miR-29b-3p mimics in IL-1β-treated CHON-001 cells, highlighting the known nutrigenomic effect of EGCG [41]. TFDG, a natural compound found in black tea, was found to increase the proliferation of IL-1β-treated chondrocytes, as well as increase ECM deposition (ACAN and Col-II) and reduce ECM degradation (decrease in MMP-13, MMP-3, and ADAMTS5). TFDG has potent anti-inflammatory actions, suppressing the phosphatidylinositol 3-kinase (PI3K)/Ak strain-transforming protein (AKT)/NF-κB and mitogen-activated protein kinase (MAPK) pathways, and antioxidative activities, accelerating Reactive Oxygen-containing Species (ROS) elimination through Nrf-2 activation. TFDG action on the cartilage of DMM rats showed lower OARSI scores and a higher expression of COL-2 and Nrf-2 compared to controls [42].

**Flavanones**, also known as dihydroflavones, lack a double bond between carbons 2 and 3, which are present in flavones and flavonols. We have found nine manuscripts regarding four molecules: baicalin, naringenin (NAR), sappanone A, wogonoside [43,44,45,46,47,48,49,50,51]. Baicalin is extracted from *Scutellaria baicalensis* Georgi, and exhibited anti-OA activities. With respect to all other natural molecules here reported, it shows a peculiar mechanism of action involving mitophagy, the particular autophagy with selective degradation of mitochondria also specifically involved in chondrocyte homeostasis, and, thus, in OA. In IL-1β-induced chondrocytes, by using different doses of baicalin as well as an autophagy inhibitor, autophagy activator, and PI3K agonist, the authors demonstrated that baicalin is a mitophagy activator by inhibiting the PI3K/AKT/mTOR pathway and activating the PINK1/Parkin and PINK1/Drp-1 pathway. Moreover, baicalin helped cell viability and caused elevated mitochondrial membrane potential and suppression of apoptosis. Thus, it is able to reduce chondrocyte injury [40]. Recently, indications of involvement of long noncoding RNA in inflammation, lipid metabolism, and other processes of OA were highlighted. In particular, lncRNA HOX transcript antisense RNA (HOTAIR) was indicated as being higher in OA chondrocytes to such an extent that it is considered a biomarker of OA in vivo. These same authors also have demonstrated that baicalin inhibits lncRNA HOTAIR expression, diminishing the protein levels of p-PI3K and p-AKT, and growing the protein levels of PTEN, OPN, and ADIPOR1 [43]. Other authors, by performing in vivo and in vitro analyses, have demonstrated that baicalin lessened OA progression in vivo and showed a chondroprotective effect in vitro by quashing chondrocyte ferroptosis, which, in turn, can reduce OA-related pain sensitivity in OA mice. This proposes for the first time baicalein as a potential therapeutic strategy for OA [45]. In Li et al. (2021), baicalein treatment in pre-osteoblast cultures decreased the differentiation of osteoblasts, inhibiting proliferation and promoting apoptosis. Furthermore, its administration to human umbilical vein endothelial cells (HUVECs) significantly reduced angiogenesis, and treatment of fibroblast-like synovial cells (FLSs) inhibited cell proliferation in in vivo experiments, showing that baicalein treatment in a DMM rat model confirmed the in vitro data, impairing subchondral bone remodeling and suppressing vascularization, thus alleviating bone sclerosis. In addition, the control of FLS proliferation reduced the progression of synovitis in OA [46]. An in vitro study on baicalin, the aglycone form of baicalein, showed that its addition promoted cell viability and reduced apoptosis in IL-1β-treated human chondrocytes. It also increased the expression of the extracellular matrix (ACAN and COL-2A) and reduced the expression of ECM-degrading enzymes (MMP-13). Li et al. (2020) associated these effects with the activation of autophagy by the upregulation of the microRNA miR-766, which targets apoptosis-inducing factor mitochondria-associated 1 (AIFM1), as indicated by overexpression and silencing experiments [47]. Moreover, in Bai et al. (2021), intra-articular injection of baicalein in a DMM rat model showed that this treatment reduced the OARSI score and decreased OA progression in a dose-dependent manner. In particular, baicalein injection led to a reduced expression of inflammatory cytokines (IL-1β and TNF-α) and ECM-degrading enzymes (MMP-13 and ADAMTS-5), as well as an increased expression of type II collagen, and inhibited the nucleotide-binding domain, leucine-rich–containing family, pyrin domain–containing-3 (NLRP-3) inflammasome activation without influencing malondialdehyde (MDA) production and superoxide dismutase (SOD) and glutathione (GSH) expressions [48]. NAR found in *Elaeodendron croceum* has significant anti-inflammatory and antioxidant effects. In Pan et al. (2022), it is shown that iron overload-induced OA is mitigated by NAR treatment. It increases the viability of chondrocytes as well as inhibiting apoptosis, and increases matrix deposition. NAR alleviated the accumulation of ROS and the lipids peroxide oxidation (LPO) process in these chondrocytes, through upregulation of Nrf-2 and HO-1. The use of the Nrf-2-specific inhibitor, ML385, partially reverses these positive effects, indicating the importance of its involvement in slowing OA progression. In vivo experiments using micro-computed tomography (μCT) and morphometric analyses of the subchondral bone showed that NAR treatment reduced the degeneration of subchondral bone structures of DMM in C57BL/6 male mice, thereby slowing OA progression [49]. Zhang et al. (2022) showed that pre-treatment with sappanone A, isolated from *Caesalpinia sappan* L., had anti-inflammatory activities, inhibiting IL-1β-dependent NO, prostaglandin E2 (PGE-2), inducible nitric oxide synthase (iNOS), cyclooxygenase-2 (COX-2), TNF-α, IL-6, and IL-8 production in chondrocytes, thus reducing both the inflammation status and oxidative stress. Furthermore, its addition suppressed also MMP-3, MMP-13, ADAMTS-4, and ADAMTS-5 expressions in these IL-1β-stimulated chondrocytes, inhibiting ECM degradation. Sappanone A exerted its effects by reducing NF-κB expression and activity, and enhancing Nrf-2/HO-1 activations [50]. Wogonoside, a flavanone extracted from *Scutellaria baicalensis* Georgi, has been shown to have antioxidant and anti-inflammatory effects. Tang et al. (2017) showed that this molecule inhibits oxidative stress and inflammation activities in IL-1β-treated mouse chondrocytes, as indicated by the reduction in iNOS and COX-2 expression, as well as its ability to inhibit hypertrophic conversion, indicated by a reduction in Col-X, runt-related transcription factor 2 (RUNX-2), and vascular endothelial growth factor A (VEGFA) expressions in a dose-dependent manner. Furthermore, wogonoside treatment increases the expression of ECM, as indicated by SRY-Box Transcription Factor 9 (SOX-9) expression, a key transcription factor in cartilage development and, consequently, Col-II and ACAN expressions, its effector genes. In vivo experiments showed how wogonoside administration reverts partial cartilage erosions and massive proteoglycan loss, confirmed by OARSI scores that were markedly higher in the OA group and returned low in the wogonoside-treated group. In addition, wogonoside administration alleviated synovitis compared to the OA group, indicating that wogonoside can protect against OA development, inhibiting proteoglycan loss, endochondral calcification, and synovia inflammation [51].

**Flavones** are obtained by dehydrogenation of flavanones, and they are the most representative class of flavonoids. We have found fifteen manuscripts on 11 molecules: acacetin, apigenin, chrysin, diosmetin (DIOS; 3′,5,7-trihydroxy-4′-methoxy flavone), luteolin, nepetin, orientin, oroxylin-A, rhoifolin (ROF), scutellarin, and velutin [52,53,54,55,56,57,58,59,60,61,62,63,64,65,66]. Acacetin (5,7-dihydroxy-4′-methoxyflavone) is the major bioactive component of the traditional Chinese medicine “Snow lotus” (*Saussurea involucrata*) and is present in some plants, such as *Robinia pseudoacacia*, *Turnera diffusa,* and *Betula pendula*. Chen et al. (2020) explored the protective effect of acacetin on cartilage, both in human and mouse specimens. They focused on one of the two factors, in their opinion, involved in post-traumatic OA: the local degradation of the extracellular matrix induced by the injury-caused pro-inflammatory cytokines expressions. They showed that, in vitro, chondrocytes pre-treated with acacetin followed by IL-1β stimulation inhibited IL-1β-induced MMP expression by targeting NF-κB pathways. Regarding in vivo studies, they explored the protective effects of acacetin on human/mouse cartilage. Taking into account the local degradation of the extracellular matrix, induced by the injury-caused pro-inflammatory cytokines, they showed the acacetin’s power to inhibit the MMP expression in in vivo experiments, and, consequently, they proposed a potential value of this flavone in the treatment of OA, since its protective effect on cartilage has been clearly demonstrated [52]. Apigenin (4′,5,7-trihydroxyflavone), a flavonoid found in fruits and vegetables with wide-ranging bioactivities, including antioxidant, anti-inflammatory, and anticancer properties, was recently studied by Ji et al. with the aim to examine the mechanism of macrophage polarization on OA-damaged chondrocytes and to reveal the probable protective effect of apigenin. Actually, they demonstrated that apigenin has a protective effect on cartilage degeneration by a significant reduction in the expression levels of IL-1β, IL-6, MMP3, and MMP13. Moreover, they also demonstrated that apigenin, with a pleiotropic effect, is able to increase and decrease some different actors with the final effect of inhibiting the M1 polarization of macrophages and promoting the M2 one [53]. The in vivo study of Estakhri et al., by considering the elevated oxidative stress markers often associated with the pathogenesis of OA, showed the effects of co-injecting apigenin and synovial membrane-derived mesenchymal stem cells (SMMSCs) in the knee joints of male rats after ACLT surgery. After three months of treatments, certain inflammation endpoints decreased and certain parameters of the antioxidant system, such as SOD, increased. Consequently, the authors concluded that apigenin might have supplementary beneficial effects in this rat model of OA, due to its possible effect on the reduction in oxidative stress and suppression of inflammation status [54]. Chrysin (5,7-dihydroxyflavone), a dietary phytochemical present in blue passion-flower (*Passiflora caerulea*), propolis, and honey, has economic value and medicinal impact, particularly as a traditional Chinese remedy. Liao et al. (2020) have demonstrated that the LPS-related inflammation response in primary rat FLSs is partially mitigated by chrysin administration, as indicated by a reduction in the expression of pro-inflammatory cytokines IL-1β and IL-18. They relate this to the interference of chrysin treatment in the NLRP-3 inflammasome, caspase-1, and the expression and activation of apoptosis-associated speck-like protein (ASP). Chrysin also exhibits some in vivo biological activities, as demonstrated in a rat knee MIA-induced OA model. By experiments of quantitative real-time polymerase chain reaction (qRT-PCR), Western blotting, enzyme-linked immunosorbent assay (ELISA), and immunofluorescence, the authors demonstrated that chrysin decreased inflammation status, reduced some other OA endpoints, inhibited NLRP-3 inflammasome activation, and ultimately alleviated the synovitis [55]. More recently, some authors, through in vivo and in vitro studies, while confirming these data, have added the important detail that chrysin also reduced the inflammatory response induced by TGF-β1 and fibrosis in FLSs with the conclusion that it can improve synovitis and fibrosis in knee OA by performing, among others, this specific mechanism of action [56]. DIOS, a natural citrus-contained flavonoid, is already known as an antioxidative, anti-inflammatory, anti-apoptotic, and anticancer compound. More recently, it was reported that it blocks DMM-induced subchondral bone loss and reduces subsequent cartilage degradation in vivo. In particular, by using a cell-based assay, some authors demonstrated that the anti-osteoclastic DIOS effect and the associated inability to affect IL-1β-induced chondrocyte hypertrophy were also due to the suppression of RANKL-induced activation of the ERK, p38, and JNK MAPK signaling pathways. Based on these findings, the authors nominate DIOS as a potential therapeutic agent against anomalous subchondral bone loss and cartilage degradation, all OA-associated phenomena [57]. Luteolin (3′,4′,5,7-tetrahydroxyflavone), discovered in *Reseda luteola*, is found in several fruits, herbs, and green vegetables, and some studies highlighted its anti-inflammatory actions. Fei et al. (2019) demonstrated how treatment with IL-1β increases the expression of inflammatory mediators (iNOS and COX-2), as well as factors implicated in cartilage matrix degradation (MMP-1, MMP-3, and MMP-13), and how this activity was partially reversed in a dose-dependent manner by luteolin administration. Furthermore, they investigated luteolin’s actions in an in vivo model of OA. In particular, the authors proposed, based on their results, that luteolin could protect rat chondrocytes by inhibiting the overexpression of inflammatory mediators, likely by blocking NF-κB activation [58]. The results reported by Zhou et al. (2022), obtained using primary murine chondrocytes, indicated that luteolin has cytoprotective effects against oxidative damage, mimicked with H_2_O_2_ treatment, and the inflammatory response, by activating the AMPK/Nrf-2 signaling pathway. Moreover, other authors focused on luteolin’s capability to protect rat chondrocytes from induced oxidative stress and to attenuate OA progression by activating the AMPK-Nrf-2 pathway, concluding that luteolin may be a useful therapeutic agent for patients with OA [59]. Nepetin (6-methoxyluteolin) is a small-molecular-weight natural flavonoid derived from *Phyla nodiflora* with several described pharmacologic effects, especially in the modulation of inflammation. Xu et al. (2021) reported an interesting study, in vitro, in vivo, and in silico, in which they described nepetin’s effects in primary mice chondrocyte cultures. They found that nepetin suppressed NF-κB activation of IL-1β-stimulated mice chondrocytes. Interestingly, the authors also performed molecular docking studies and reported that nepetin can exert a favorable interaction with the p65-binding site against an antagonist for the same site. In a DMM OA model in mice, the same researchers reported that oral administration of nepetin daily for 14 days improved the OA course, showing protective and therapeutic effects. According to the OARSI criteria, the nepetin-treated mouse group showed a score significantly lower than the OA group. They concluded by proposing nepetin as a potential prophylactic treatment option for OA [60]. Orientin, a C-glycosyl dietary flavone contained also in red tea leaves, is already known to be anti-inflammatory with bioactive properties. More recently, some authors, based on their results in vitro and in vivo, have asserted that it is able also to attenuate OA manifestations and progression. In particular, data obtained by in vitro experiments have demonstrated that orientin induces a significant suppression in IL-1β-mediated pro-inflammatory mediators. Moreover, the in vivo studies indicate that it annulled DMM surgery-induced cartilage degradation in mice. Lastly, by additional mechanistic studies, they conclude that orientin suppressed OA development by downregulating activation of NF-κB, by activating the Nrf2/HO-1 axis and Sirt-6 signaling pathway [61]. Oroxylin-A, found in *Oroxylum indicum*, has anti-inflammatory, anti-apoptotic, and anti-tumor effects. Particularly interesting are the results reported by Chen et al. (2021a), obtained in vitro on IL-1β-induced primary human chondrocytes. Oroxylin-A displayed no cytotoxic activity, decreased cell inflammation by inhibiting NF-κB activation, and lessened the hypertrophic changes by inhibiting the Wnt/β-catenin signaling pathway, as demonstrated by in silico molecular docking data. In terms of in vivo results on oroxylin-A, its treatment ameliorated the course of OA in a surgically induced OA mouse model, as shown in comparative X-ray images in which its chondroprotective effects were evident. Moreover, the authors, based on an oroxylin-A therapeutic dose for mice, calculated the dose for humans, demonstrating that the amount of stem bark of *Oroxylum indicum* utilized was compatible with a normal diet intake. These data also suggest that oroxylin-A, achieved via dietary intake, could be a promising therapeutic molecule in OA prognosis [62]. ROF is a molecule found in *Plumula Nelumbinis* and, like other flavones, it inhibits the expressions of SASP factors and, as a result, the senescence phenotype in chondrocytes. Moreover, molecular docking and knock-down analyses showed that ROF could bind to Nrf-2 to suppress the NF-κB pathway, as demonstrated by Chen et al. (2022a). They also reported that in an ACLT rat model, ROF greatly ameliorated the OA condition, via the Nrf-2/NF-κB axis. In particular, safranin O, HE, and Masson’s staining indicated that its administration could significantly reduce OA’s pathological changes, such as cartilage destruction and synovial inflammation. Furthermore, immunohistochemistry showed that ROF could reduce inflammatory cytokine (IL-6)- and ECM-degrading (MMP-13) factor expressions, as well as increase the expression of ECM molecules (ACAN) and antioxidative factors (Nrf-2) in the joints of the OA group, confirming the results of the in vitro study and suggesting that ROF could be a potential therapeutic molecule for OA prognosis [63]. Scutellarin is a flavonoid contained in the Chinese traditional herb *Erigeron breviscapus*. In an ATDC5 cell line in vitro, Yang et al. (2022b), based on the results obtained, hypothesized that scutellarin regulates OA by inhibiting the PI3K/AKT/mTOR signaling pathway. In vivo data showed that scutellarin significantly reduced cartilage damage in two different mouse models (DMM and ovariectomy—OVX—mice) by deterring the upregulation of some inflammatory factors. SCU alleviated the subchondral bone damage and ameliorated articular cartilage degeneration in OVX mice, as well as in DMM-induced OA mice, interfering with NF-κB and MAPK pathways [64]. More recently, Ju et al. (2021) demonstrated, using an IL-1β-activated human osteosarcoma cell line (SW1353), that scutellarin inhibits the IL-1β-activated MAPK/NF-κB signaling pathway, as well as regulating the PI3K/AKT/mTOR signaling pathway, as has already been demonstrated. They hypothesize that scutellarin regulates OA in vitro through these pathways [65]. Velutin is a glycoside extracted from mistletoe (*Viscum album*) with protective effects against a variety of diseases and the property of inhibiting the expression of some pro-inflammatory cytokines. Wang et al. (2022) demonstrated that velutin can inhibit chondrocyte inflammation and prevent the development of osteoclasts and bone resorption in vitro, via the p38-MAPK signaling pathway. Velutin’s protective effects were also seen in a mouse OA model (DMM) in both articular cartilage degeneration and subchondral bone loss; it inhibits the increase of mature osteoclasts and bone resorption triggered by RANKL [66].

**Flavonols** are polyphenols that comprise a major class of the flavonoid family. Their biosynthesis is stimulated by light, so they mainly accumulate in the outer tissues, such as the skin and leaves, of fruits and vegetables. Flavonols are interesting for their biological activity, such as being antioxidant, antimicrobial, hepatoprotective, and anti-inflammatory, and, more recently, they have also been studied as potential anticancer agents. In our research, we found twelve manuscripts on six molecules: astilbin, galangin (3,5,7-trihydroxyflavone, Gal), hyperoside, icariin, quercetin (Que), and rutin [67,68,69,70,71,72,73,74,75,76,77,78]. Astilbin is a flavonol identified in *Dimorphandra mollis*, *Hypericum perforatum,* and other natural plants, and it is also extracted from grapes. It shows a lot of pharmacological activities, such as being antioxidant, anti-inflammatory, and anti-tumor. Sun et al. (2020) showed an anti-inflammatory effect of astilbin in LPS-induced human chondrocytes. Its treatment decreased the expression of PGE-2, COX-2, and iNOS, as well as TNF-α and IL-6, and inhibited NF-κB activation in human OA chondrocytes stimulated with LPS, used for mimicking the inflammatory environment. Astilbin is also involved in the maintenance of cartilage ECM. In fact, the pre-treatment of astilbin in human OA chondrocytes decreases LPS-induced expression of MMP-13 and ADAMTS-5 and increases the expression of Col-II and ACAN. Toll-like receptor (TLR)-4 signaling is an upstream cascade that influences NF-κB activation in OA development. Therefore, as Sun et al. (2020) have demonstrated using docking analysis, astilbin is able to affect the TLR-4/MD-2 axis. In particular, the myeloid differentiation factor 2 (MD-2) inhibitory pocket is shown specifically to be bound by astilbin, which interferes with the formation of the TLR-4/MD-2 complex. Furthermore, they have also evidenced the protective ability of astilbin in the OA process in vivo, using a DMM-induced OA mouse model, obtained through surgery. The treatment with astilbin partially reversed cartilage calcification and erosion. The OARSI score in the OA DMM group was significantly higher than that of the sham group, as confirmed by safranin O staining. In the astilbin group, the OARSI score was lower compared to the OA DMM group, suggesting the beneficial effects of astilbin in countering the development of OA in vivo [67]. Gal is a flavonoid extracted from *Alpinia officinarum* Hance, which has shown potent anti-inflammatory and antioxidative activities in several cell systems. In Lin et al. (2022), Gal treatment showed an increased ECM production, as well as a reduction in MMP and ADAMTS, which are implicated in its degradation. This appears to be due to the induced expression of proline- and arginine-rich end leucine-rich repeat protein (PRELP), a heparin/heparan sulphate-binding protein expressed in cells of collagen-rich tissues and under-expressed in OA patients. This is confirmed by the expression of si-RNA through PRELP mRNA, using a lentivirus, which showed the partial reversion of Gal activities. Using the DMM OA model in rats, Lin et al. (2022) showed that Gal administration can ameliorate OA progression, increasing ECM production and reducing its degradation. Furthermore, the OARSI score confirms this evidence [68]. Hyperoside is a bioactive flavonol glycoside, isolated from *Epimedium brevicornum* and *Hypericum perforatum*, that is abundant in various fruits, vegetables, and medicinal plants, and it has been shown to exert anti-inflammatory and anti-apoptotic functions. In Sun et al. (2021), hyperoside repressed the IL-1β-induced NF-κB-dependent inflammatory response in chondrocytes and attenuated IL-1β-induced ECM degradation. Hyperoside treatment of chondrocytes attenuated IL-1β-induced oxidative stress and apoptosis through the Nrf-2/ROS/BCL-2-associated X protein (BAX)/B-cell lymphoma extra-large protein (BCL-XL) axis. Moreover, the knockdown of Nrf-2 partially abolished hyperoside’s effects on reducing the ROS level and apoptosis, indicating that hyperoside exerts its protective role against IL-1β-induced apoptosis in chondrocytes via Nrf-2. In a DMM OA model, the intraperitoneal injection of hyperoside inhibited the loss of glycosaminoglycan and cartilage destruction, as evaluated by hematoxylin-eosin, safranin O staining/fast green and toluidine blue staining, and confirmed by the OARSI score [69]. Icariin (ICA) is an 8-isopentene flavonol, which has been isolated from several species of plants belonging to the genus *Epimedii Folium*. It has been reported that ICA has broad pharmacological effects, such as anti-inflammation, anticancer, and bone-protective effects. In Chen et al. (2022b), ICA promotes the proliferation of chondrocytes, affecting the ECM environment, including via the excessive production of MMP-3 and degradation of Col-II, and antagonizing IL-1β-induced chondrocyte degeneration. Furthermore, ICA increases the expression of autophagy proteins, including microtubule-associated protein 1A/1B light chain 3 (LC3) and Beclin1, which are commonly used as biomarkers of autophagy. ICA treatment also increases the expression of Unc-51-like autophagy-activating kinase (ULK1), a serine/threonine kinase, essential in autophagy processes. ICA inhibits IL-1β-induced phosphorylation of PI3K, AKT, and mTOR, and attenuates the excessive production of MMP-3 and promotes the production of Col-II. Silencing ULK1 reverses the protective effect of ICA in OA chondrocytes, demonstrating that ULK1 and the autophagy process are essential for ICA’s mechanism of action [70]. In Wang et al. (2021), ICA induced the expression of an lncRNA, Cytoskeleton Regulator RNA (CYTOR), implicated in age-related articular cartilage degradation. Indeed, CYTOR is significantly downregulated in chondrocytes stimulated with IL-1β. Overexpression of CYTOR inhibited apoptosis in IL-1β-stimulated chondrocytes and promoted its viability. In OA models, ICA and CYTOR downregulated the levels of inflammatory cytokines (IL-6, IL-8, and TNF-α). The protective effect of ICA on chondrocytes was reversed with CYTOR knockdown [71]. In Wang et al. (2020b), ICA exerted its anti-inflammation activity by downregulating the expression of HIF-2α, MMP-9, and ADMTS-5, induced by TNF-α in chondrocytes. Furthermore, ICA treatment downregulated the ratios of p-IKKα/β/IKKα/β (IκB kinase), p-IκB/IκB (nuclear factor of kappa light polypeptide gene enhancer in B-cells inhibitor), and p-NF-κB/NF-κB, and inhibited the NF-κB nucleic translocation. The anti-inflammatory effect of ICA was also investigated in vivo in an articular-cartilage-defect mouse model. A slow-release and absorbable gelatin was implanted with or without ICA into the defects and the ECM synthesis was tested. The ECM synthesis was significantly higher in the group treated with alginate–gelfoam complexes containing ICA than in the untreated control. In addition, the expressions of NF-κB and hypoxia-inducible factor (HIF)-2α were significantly decreased in the mice implanted with the alginate–gelfoam complexes containing ICA. Thus, ICA significantly inhibited cartilage degradation, inflammation, and NF-κB/HIF-2α signaling pathways in vivo [72]. In Tang et al. (2021), ICA induced autophagy in chondrocytes in an OA animal model and decreased their apoptotic rate in a dose-dependent manner. This effect was mediated by the PI3K/AKT/mTOR signaling pathway, which was markedly suppressed by icariin. Furthermore, following treatment with icariin, the severe pathological state in OA cartilage tissues was substantially alleviated, and this was accompanied by activated autophagy and inhibited PI3K signaling in the cartilage tissues of the OA animal model [73]. Recently, ICA’s therapeutic potential was also shown in combination with other compounds. In fact, an ICA-conditioned serum pooled with thiolated chitosan, a known material used in tissue engineering, was observed to be able to attenuate cartilage injury in rabbit knees with osteochondral defects [74]. Quercetin (Que) belongs to the family of flavonols and is one of the most abundant flavonoids assumed with diet. It has many forms in plants and generally functions as a pigment that gives color to a multitude of fruits and vegetables. Lv et al. (2022) showed that QUE inhibited MMP-9, MMP-13, and ADAMTS-5 expressions and increased SOX9, Col-II, and ACAN expressions. Moreover, QUE induced autophagy of chondrocytes, enhancing the expression of LC3BII/I, downregulating the expressions of Ras Homolog Enriched in Brain 1 (RHEB) and P62, and inhibiting the phosphorylation of mTOR and ULK1 in chondrocytes. Indeed, QUE regulates autophagy of KOA cells through the Tuberous Sclerosis Complex 2 (TSC2)/RHEB/mTOR signaling pathway, thus silencing TSC2, although shTSC2 reversed the therapeutic effect of QUE. The same authors showed, in a rat model of knee OA, how the joint tissue appeared with irregularities, cracks, and even loss of articular cartilage, but in the group where the QUE was administrated, it was relieved, as indicated by HE and safranin O staining [75]. Recently, two preclinical studies took into consideration QUE’s anti-osteoarthritis effect in a rat model, finding a good effect on the inhibition of OA development. The first study showed that QUE acts through modification of the AMPK/mTOR signaling pathway [76]. Moreover, the second one demonstrated that high-dose quercetin was able to reverse the levels of most inflammatory cytokines and growth factors in synovial fluid/serum and, consequently, was capable of contrasting subchondral trabecular bone degradation [77]. Rutin is a glycoside of QUE found in many plants and fruits, such as buckwheat, apricots, cherries, grapes, grapefruit, etc. Rutin has been reported to have many biological activities targeting various inflammatory proteins, thus improving inflammatory conditions. In Chen et al. (2021b), rutin acted to inhibit the levels of inflammatory factors such as IL-6 and TNF-α, tumor necrosis factor receptor-associated factor 6 (TRAF-6), and B-cell leukemia/lymphoma 2 protein (BCL-2), and it inhibited MAPK and NF-κB pathways. Moreover, rutin suppresses in human chondrocytes AGE-induced levels of COX-2, PGE-2, and iNOS, and NO at both protein and mRNA levels, showing a nutrigenomic effect. The insult of AGEs in the cartilage of joints leads to an imbalance between the anabolism, catabolism, and ECM molecules. In AGEs-induced chondrocytes, rutin exerts chondroprotective activity by inhibiting the degeneration of ACAN and Col-II, alleviating levels of ADAMTS and MMP. Furthermore, in vivo experiments show that rutin had potential binding with TRAF-6 and BCL-2, evaluated by in silico molecular docking analysis. In an in vivo model of OA induced by DMM, treatment with rutin corrected cartilage defects, with a significant decrease in OARSI scores [78].

**Isoflavones** are phytoestrogens with potent estrogenic activity. They have chemoprotective effects and can be used as an alternative therapy for a wide range of hormonal disorders. In our research, we found eight manuscripts on seven molecules: biochanin A (BCA), calycosin, caviunin glycoside (CAFG), S-equol, glabridin, neobavaisoflavone (NBIF), and ononin [79,80,81,82,83,84,85,86]. BCA is an isoflavone isolated from *Astragalus membranaceus*, with known protective effects on bone loss. In the study of He et al. (2023b), the researchers showed how iron overload increased chondrocyte ferroptosis (iron-dependent programmed cell death) and cartilage degeneration, enhancing Col-II degradation and inhibiting its synthesis. BCA is able to directly reduce intracellular iron concentrations by inhibiting TfR1 and promoting FPN, but also by activating Nrf2 pathways, increasing the scavenging of ROS, as well as preventing lipid peroxidation. These activities reduce the severity of OA, as indicated by the OARSI score from safranin O/fast green staining in a DMM mouse model of OA with iron overload. Additionally, they ameliorate subchondral bone degeneration, reducing bone loss, as indicated by a quantitative analysis of micro-CT [79]. Calycosin is an isoflavone extracted from the root of Radix astragali, with anti-allergy, anti-inflammation, and anti-tumor activities. In Guo et al. (2022), calycosin was found to protect chondrocytes from IL-1β-induced apoptosis. Moreover, calycosin upregulated Col-II and ACAN, and downregulated ADAMTS5 and MMP-13, preventing IL-1β-caused ECM degradation. In addition, calycosin in IL-1β-stimulated chondrocytes inhibits the activation of the PI3K/AKT/FoxO1 pathway. FoxO1 is a transcription factor that regulates bone formation, inducing cartilage loss, and calycosin is able to inhibit FoxO1 transcription activity [80]. In Shi et al. (2022), calycosin inhibited the phosphorylation of the PI3K/AKT pathway and reduced apoptosis in mouse chondrocytes in a dose-dependent manner. Furthermore, calycosin acted as an anti-inflammatory agent. It can inhibit the expression of inflammatory genes induced by IL-1β, such as IL-6, iNOS, TNF-α, and COX-2, showing its nutrigenomic property. Moreover, calycosin inhibited p65 phosphorylation in IL-1β-treated chondrocytes, showing the involvement of NF-κB signaling in its mechanism of action and preventing the development of OA. Regarding the in vivo studies on isoflavonoids, Shi et al. (2022) demonstrate also that calycosin attenuated IL-1β-induced ECM damage. Calycosin downregulates the expression of matrix degradation proteins, such as MMP-3 and MMP-13. Furthermore, calycosin restored the IL-1β-induced downregulation of Col-II and ACAN, improving cartilage pathology and reducing the OARSI score in DMM mice [81]. CAFG is an isolated compound from *Dalbergia sissoo* DC, an Indian medicinal plant. Kothari et al. (2022) demonstrated that in an in vitro model of osteoarthritis in which rat chondrocytes were stimulated with IL-1β, CAFG decreased the IL-1β induced inflammation effects. In particular, CAFG reduced ROS generation after IL-1β induction. Furthermore, Kothari et al. (2022) demonstrate that CAFG decreased inflammation serum biomarkers such as MMPs, which degraded the matrix protein of cartilage and released carboxy-terminal telopeptides of type II collagen (CTX-II) and cartilage oligomeric matrix protein (COMP) in in vivo studies. CAFG also protected against IL-1β-induced chondrocyte death [82]. S-equol (7-hydroxy-3-(40-hydroxyphenyl)-chroman) is an isoflavone derived from soy; it is a daidzein metabolite. S-equol is well known among all soy isoflavones because of its strong estrogen receptor-binding capacity and its antioxidative activities. In Huang et al. (2021), rat primary chondrocytes were treated with SNP to mimic OA. S-equol treatment protected chondrocytes from SNP-induced apoptosis and matrix degradation by decreasing the expressions of MMPs such as MMP-2, MMP-3, MMP-9, and MMP-13. Moreover, S-equol can decrease the expression of p53 induced by SNP in primary chondrocytes. In particular, S-equol activates phosphorylation of AKT and mouse double minute 2 (MDM2), which is an upstream regulator of p53. Indeed, the mechanism by which S-equol protects chondrocytes from SNP-induced matrix degradation and apoptosis is through the PI3K/AKT pathway [83]. Glabridin is a type of isoflavone extracted from a plant called *Glycyrrhiza glabra*, with strong antioxidant effects on free radicals, similar to vitamin E, and with marked activity in pathological conditions associated with free radical oxidation, such as atherosclerosis and cell aging. In Dai et al. (2021), glabridin enhanced the activities of antioxidant enzymes and scavenged free radicals in OA chondrocytes, as glabridin treatment significantly increased the activities of catalase and superoxide dismutase. Through in vitro experiments, the same authors showed that glabridin effectively reduced the apoptosis ratio of OA chondrocytes. Glabridin is also able to stimulate the autophagy of human OA chondrocytes; this was confirmed by electron microscopy and the increase in LC3 protein. Indeed, glabridin can inhibit the expression of mTOR and enhance autophagy in human OA chondrocytes. Furthermore, glabridin is able to restore the decrease in ECM structural molecules including Col-II and ACAN in in vitro studies. Furthermore, in in vivo experiments, following ACLT surgery, articular injection of glabridin decreased the loss of main components in the extracellular matrix. Histological sections of the cartilage tissue, stained with HE, safranin O–fast green, and Alcian Blue, confirmed how articular cartilage erosion and reduced proteoglycan loss were significantly inhibited by glabridin; this evidence was confirmed by the OARSI grading system [84]. NBIF is an isoflavone isolated from *Psoralea corylifolia* L. that showed anti-inflammatory and antioxidative activities. In Bai et al. (2022), this molecule was able to reduce the release of pro-inflammatory cytokines in IL-1β-induced rat chondrocytes, inhibiting the NF-kB pathway. Its administration was able to alleviate OA in a DMM rat model, reducing also here pro-inflammatory cytokines production, as well as oxidative stress, as indicated by MDA, SOD, and CAT indicators [85]. Ononin is an isoflavone component present in traditional Chinese medicines, such as *Astragalus membranaceus*, *Glycyrrhiza uralensis*, *Hedysarum,* and *Pueraria lobata*. Ononin has an antioxidant and anti-inflammatory capability and may modulate cell proliferation. In Xu et al. (2022), ononin inhibited the IL-1β-induced expression of the cytokines TNF-α and IL-6, as markers of inflammation in primary chondrocytes. These pro-inflammatory factors (TNF-α and IL-6) induce the production of MMPs (enzymes that induce articular cartilage degradation) and inhibit the synthesis of ECM. They have demonstrated also that ononin reduced the MMP-13 expression and increased Col-II expression, restoring finally the ECM degradation. Furthermore, the mechanism by which ononin regulated the inflammatory response and ECM degradation included the regulation of the MAPK and NF-κB signaling pathways. In fact, ononin inhibits, in a dose-dependent manner, the phosphorylation of extracellular signal-regulated kinase (ERK), c-Jun N-terminal kinase (JNK), and p38-MAPK in IL-1β-stimulated chondrocytes, highlighting the inhibition of the IL-1β-activated MAPK pathway. Moreover, ononin decreases the expression of p-IκBα in IL-1β-activated chondrocytes, thus reducing the release of p65, which translocates into the nucleus and upregulates the expression of secreted inflammatory factors: in this way, ononin is able to regulate NF-κB signaling pathways. Thus, ononin may be an effective drug for the treatment of OA [86].

**Table 1:** List of flavonoids implicated in cartilage metabolism and activities in OA disease. The arrow↓indicates a reduction/down regulation while the arrow↑indicates increase/up regulation.

Flavonoid Subgroup	Compound	Study Type	In Vitro/In Vivo Model	Doses	Analysed Pathways/Process	Effects of Administration	Ref
Anthocyanins	Cyanidin (C3G)	In vivo	CIA model in Sprague Dawley (SD) rats	25 mg/kg, twice per week for six weeks, via tail vein	cytokine concentrations; SIRT-6 pathway;	↓ Pro-inflammatory cytokines; ↑ IL-10; Alteration of white cell sub-populations.	[31]
		In vitro	Human adipose MSC;	25 μM, 50 μM, 100 μM, 200 μM	-	↑ Chondrocyte differentiation.	[30]
	Delphinidin	In vitro	Human adipose MSC;	25 μM, 50 μM, 100 μM, 200 μM	-	↓ Adipocytic differentiation; ↑ Chondrocyte differentiation.	
	Malvidin	In vitro	Human adipose MSCs;	25 μM, 50 μM, 100 μM, 200 μM	BMP pathway	↑ OB differentiation.	
	Pelargonidin (PG)	In vitro	Mouse chondrocytes exposed to IL-1β (10 ng/mL)	0.5 μM, 1 μM, 5 μM, 10 μM, 20 μM, 40 μM, 80 μM, 160 μM	cytokine concentrations; NF-kB pathway	↓ Pro-inflammatory cytokines; ↑ ECM production; ↓ ECM degradation; ↓ Degradation of cartilage in mouse OA model	[32]
		In vivo	DMM in Male C57BL/6 mice	10 mg/kg/day; 20 mg/kg/day; orally			
	Procyanidin B2 (PCB2)	In vitro	SD rat chondrocytes exposed to IL-1β (10 ng/mL)	5 μM, 10 μM, 20 μM, 40 μM, 80 μM	Apoptosis; Nrf-2 pathway; NF-κB pathway; p16(INK4A)/p21(CIP) expression	↓ Chondrocyte senescence; ↓ ECM degradation; ↓ Pro-inflammatory cytokines;	[33]
		In vivo	Destabilization of the medial meniscus (DMM) in male SD rats	40 mg/kg twice per week for six weeks			
Chalcone	Butein	In vitro	OA chondrocytes treated with IL-1β (1 ng/mL)	2.25 μM, 4.5 μM, 9 μM, 18 μM, 36 μM	mTOR Pathway; IL-6 concentrations; Autophagy;	↓ IL-6; ↑ Autophagy; ↓ mTOR.	[34]
	Cardamonin	In vitro	Human chondrocyte line (CHON-001) exposed to IL-1β (10 ng/mL)	1 μM, 3 μM, 10 μM, 30 μM, 100 μM	Apoptosis; cytokine concentrations; Nrf2 pathway;	↓ ECM degradation; ↓ Apoptosis; ↓ Pro-inflammatory cytokines.	[35]
	Isoliquiritigenin (ISL)	In vitro	Human subconjunctival fibroblasts or mouse peritoneal macrophages exposed to angiotensin (1 μg/mL)	1.25 μM, 2.5 μM, 5 μM, 10 μM, 20 μM, 40 μM	NF-κB pathway; cytokine concentrations; PPAR-γ COX-2;	↓ Pro-inflammatory cytokines; ↓ PPAR-γ degradation; ↓ Fibrosis	[36]
		In vivo	Anterior cruciate ligament transection (ACLT) in C57BL/6 J mice	10 mg/kg/day; 20 mg/kg/day; 40 mg/kg/day; 80 mg/kg/day	RANKL pathway;	↓ ECM degradation; ↓ Osteoclastogenesis; ↓ TGF-β release in subchondral bone; ↓ Angiogenesis in subchondral bone	[37]
	Licochalcone A (Lico A)	In vitro	chondrocytes of male C57BL/6 mice exposed to LPS (1 μg/mL)	5 μM, 25 μM, 50 μM; 100 μM	Apoptosis; Nrf-2 pathway; NF-κB pathway; cytokine concentrations;	↓ ECM degradation; ↓ Apoptosis; ↓ Pro-inflammatory cytokines;	[38]
		In vivo	DMM in male C57BL/6 mice	intragastric administration of 10 mg/kg/day for 8 weeks			
	Safflower yellow (SY)	In vitro	Rat chondrocytes exposed to TNF-α (no concentration)	1 μM, 5 μM, 10 μM, 20 μM	Apoptosis; NF-κB pathway; ERK pathway	↓ ECM degradation; ↓ Pro-inflammatory cytokines; ↓ Degradation of cartilage in rat OA model	[39]
		In vivo	ACLT in SD rats	1 mM SY (in sterile saline solution; 200 μL) injected intra-articular route in the knee			
	Xanthohumol (XN)	In vitro	chondrocytes of male C57BL/6 mice exposed to IL-1β (10 ng/mL)	10 μM, 25 μM, 50 μM, 100 μM, 200 μM	cytokine concentrations; NF-κB pathway; Nrf-2 pathway;	↓ pro-inflammatory cytokines; ↓ ECM degradation;	[40]
		In vivo	DMM in Male C57BL/6 mice	40 mg/kg/day intragastric administration for 8 weeks			
Flavanols	epigallocatechin (EGCG)	In vitro	Human chondrocyte line (CHON-001) exposed to IL-1β (5 ng/mL)	20 μM, 50 μM	Apoptosis; ERK pathway	↓ pro-inflammatory cytokines; ↓ ECM degradation; ↑ PTEN	[41]
	Theaflavin-3,3’-Digallate (TFDG)	In vitro	Rat chondrocytes exposed to IL-1β (10 ng/mL)	1 μM, 10 μM, 20 μM, 40 μM, 80 μM, 120 μM	Nrf-2 pathway; NF-κB pathway; ERK pathway; JNK pathway; P38 pathway;	↓ Pro-inflammatory cytokines; ↓ Oxidative stress; ↓ ECM degradation.	[42]
		In vivo	DMM in male SD rats	100 μL of saline solution, containing 4 mM TFDG, into knee joint every 2 days for 6 weeks			
Flavanones	Baicalein	In vitro	rat chondrocytes exposed to IL-1β (10 ng/mL)	10 μM, 20 μM, 40 μM	mTOR pathway; mitophagy	↓ Apoptosis; ↑ Autophagy; ↑ Mitophagy;	[43]
		In vitro	Human OA chondrocytes	50 ng/mL	AKT pathway	↓ Apoptosis; ↓ Pro-inflammatory cytokines; ↑ Anti-inflammatory cytokines;	[44]
		In vitro	Human OA chondrocytes exposed to IL-1β (10 ng/mL); Mouse chondrocytes;	1 μM, 5 μM, 10 μM	Nrf-2 pathway;	↓ Apoptosis; ↓ Ferroptosis; ↓ ECM degradation; ↓ Oxidative stress;	[45]
		In vivo	DMM in C57BL/6 mice	1 mg/kg once weekly for 10 weeks			
		In vitro	SD rat Osteoblasts; SD rat Fibroblast-like synovial cells	2.5 μM, 5 μM, 10 μM, 20 μM, 50 μM	osteogenic markers;	↓ Subchondral ossification; ↓ FLS proliferation; ↓ Angiogenesis of subchondral bone;	[46]
		In vivo	DMM in male SD rats	intra-articular injection of 0.1 mg (50 µl) once weekly for 10 weeks			
		In vivo	DMM in male SD rats	0.8 μg/L (50 μL), 1.6 μg/L (50 μL), 3.2 μg/L (50 μL), once a week for 6 weeks	Oxidative stress; cytokine concentrations;	↑ ECM production; ↓ ECM degradation; ↓ Pro-inflammatory cytokines.	[48]
		In vitro	human OA chondrocytes exposed to IL-1β (10 ng/mL)	20 μM	Apoptosis; Autophagy;	↓ Apoptosis; ↓ ECM degradation; ↑ Autophagy;	[47]
	Naringenin	In vitro	Mouse chondrocytes exposed to IL-1β (10 ng/mL)	5 μM, 10 μM, 20 μM, 40 μM, 80 μM	Apoptosis; Oxidative stress; Nrf-2 pathway;	↓ Apoptosis; ↓ ECM degradation;	[49]
		In vivo	DMM in Male C57BL/6 mice	100 mg/kg/day; 60 mg/kg/day by oral gavage			
	Sappanone A	In vitro	Human OA Chondrocytes from patients exposed to IL-1β (10 ng/mL)	5 μM, 10 μM, 20 μM	Nrf-2 pathway; Oxidative stress; NF-κB pathway;	↓ ECM degradation; ↓ pro-inflammatory cytokines	[50]
	Wogonoside	In vitro	mice primary chondrocyte exposed to IL-1β (10 ng/ml)	12.5 μM, 25 μM, 50 μM, 100 μM, 200 μM	NF-κB pathway; Oxidative stress; cytokine concentrations ERK pathway;	↓ Pro-inflammatory cytokines; ↓ Chondral hypertrophy; ↓ ECM degradation ↓ Angiogenesis of subchondral bone;	[51]
		In vivo	DMM in C57BL/6 mice	40 mg/kg/day for 8 weeks			
Flavones	Acacetin	In vitro	Primary murine articular chondrocytes exposed to IL-1β (10 ng/mL)	3.125 μM, 6.25 μM, 12.5 μM, 25 μM, 50 μM, 100 μM, 200 μM	NF-κB pathway; ERK pathway; JNK pathway; P38 pathway; cytokine concentrations	↓ ECM degradation; ↓ pro-inflammatory cytokines	[52]
		In vivo	ACLT in C57BL/6 mice	3.125 μM, 6.25 μM intra-articular injections twice per week for six weeks			
	Apigenin	In vitro	Mouse chondrocytes, Raw 264.7 cell line exposed to LPS (100 ng/mL)	10 μM	Apoptosis; mTOR pathway; M1 to M2 macrophage transition;	↓ Pro-inflammatory cytokines; ↓ Apoptosis; Alteration of Macrofage sub-populations.	[53]
		In vivo	ACLT in C57BL/6 mice	30 mg/kg/day gavaged daily for 4 weeks			
		In vivo	ACLT in male SD rats	0.1 μM, 0.3 μM (50 μL) intra-articular injections once a week for 3 weeks	Oxidative stress; cytokine concentrations	↓ Pro-inflammatory cytokines; ↑ ECM production; ↓ ECM degradation;	[54]
	Chrysin	In vitro	Rat FLSs exposed to LPS (5 μg/mL)	1 μg/mL, 2 μg/mL, 2.5 μg/mL, 5 μg/mL, 10 μg/mL, 20 μg/mL, 40 μg/mL	cytokine concentrations; NLRP-3 Inflammasome Activation	↓ Pro-inflammatory cytokines; ↓ MIA-induced synovitis	[55]
		In vivo	MIA-induced OA in SD rats	10 mg/kg/day intragastric administration for 14 days			
		In vivo	ACLT in SD rats	10 mg/kg/day; 25 mg/kg/day; intragastric administration for 28 days	NLRP-3 Inflammasome Activation	↓ synovial fibrosis; ↓ Pro-inflammatory cytokines; ↓ synovitis;	[56]
	Diosmetin	In vitro	BMM cells	1.25 μM, 2.5 μM, 5 μM, 10 μM, 20 μM	ERK pathway; JNK pathway; P38 pathway;	↓ osteoclastogenesis; ↓ subchondral bone loss	[57]
		In vivo	DMM in C57BL/6 mice	1 mg/kg/day; 5 mg/kg/day; for 30 or 60 days			
	Luteolin	In vitro	Rat chondrocytes exposed to IL-1β (10 ng/mL)	25 μM, 50 μM, 100 μM, 200 μM	NF-κB pathway; Oxidative stress; cytokine concentration	↓ ECM degradation; ↓ Pro-inflammatory cytokines	[58]
		In vivo	MIA-induced OA in SD rats	10 mg/kg/day for 45 days			
		In vitro	murine chondrocytes exposed to H_2_O_2_ (300 μM)	1 μM, 5 μM, 10 μM, 20 μM, 50 μM	Nrf-2 pathway; Apoptosis; Oxidative stress; cytokine concentrations	↓ H_2_O_2_-Induced Apoptosis; ↓ Pro-inflammatory cytokines	[59]
		In vivo	DMM in C57BL/6 mice	10 mg/kg/day; intragastric administration for 8 weeks			
	Nepetin	In vitro	murine chondrocytes exposed to IL-1β (10 ng/mL)	2.5 μM, 5 μM, 10 μM, 20 μM	Oxidative stress; cytokine concentrations; NF-κB pathway;	↓ Pro-inflammatory cytokines; ↓ Oxidative stress; ↓ ECM degradation;	[60]
		In vivo	DMM in C57BL/6 mice	20 mg/kg/day for 14 days (oral administration)			
	Orientin	In vitro	Mouse chondrocytes exposed to IL-1β (10 ng/mL)	10 µM, 25 µM, 50 µM, 100 µM, 200 µM.	Oxidative stress; cytokine concentrations; NF-κB pathway; SIRT-6 pathway;	↓ Pro-inflammatory cytokines; ↓ Oxidative stress; ↑ ECM production; ↓ ECM degradation;	[61]
		In vivo	DMM in C57BL/6 mice	30 mg/kg; once every 2 days for eight weeks			
	Oroxylin A	In vitro	human chondrocytes exposed to IL-1β (10 ng/mL)	2.5 μM, 5 μM, 10 μM, 20 μM, 50 μM	NF-κB pathway; Oxidative stress;	↓ Pro-inflammatory cytokines; ↓ Oxidative stress; ↓ ECM degradation; ↓ Chondrocyte hypertrophy	[62]
		In vivo	DMM in C57BL/6 mice	10 mg/kg/day by oral gavage for 4 weeks			
	Rhoifolin (ROF)	In vitro	Rat primary chondrocytes	5 μM, 10 μM, 20 μM, 100 μM, 200 μM, 400 μM	NF-κB pathway; Nrf-2 pathway; cytokine concentrations;	↑ ECM production; ↓ ECM degradation; ↓ Pro-inflammatory cytokines	[63]
		In vivo	ACLT in male SD rats	20 mg/kg; intragastric administration once 2 days for 8 weeks			
	Scutellarin	In vitro	SW1353 cell line exposed to IL-1β (10 ng/mL)	5 μM, 10 μM, 20 μM, 40 μM, 80 μM, 100 μM	mTOR pathway; cholesterol-related proteins;	↑ ECM production; ↓ ECM degradation; ↓ Pro-inflammatory cytokines	[64]
		In vitro	Mouse ATDC5 cell line exposed to IL-1β (10 ng/mL)	1.56 μM, 3.12 μM, 6.25 μM, 12.5 μM, 25 μM, 50 μM, 100 μM, 200 μM	NF-κB pathway; ERK pathway; JNK pathway; P38 pathway; Apoptosis;	↓ ECM degradation; ↓ Apoptosis	[65]
		In vivo	DMM in C57BL/6 mice	25 μM, 50 μM, twice a week for 12 weeks			
		In vivo	OVX in C57BL/6 mice	25 mg/kg; 50 mg/kg; intraperitoneal injection twice a week for 8 weeks.			
	Velutin	In vitro	murine chondrocytes exposed to IL-1β (10 ng/mL)	32 μM	Oxidative stress; cytokine concentrations; osteoclastogenesis; P38 pathway;	↑ ECM production; ↓ ECM degradation; ↓ Pro-inflammatory cytokines; ↓ Osteoclastogenesis; ↓ Subchondral bone deterioration;	[66]
		In vivo	DMM in C57BL/6 mice	32 μM (5 μL) once a week for 8 weeks			
Flavonols	Astilbin	In vitro	Primary human chondrocyte exposed to LPS (1 μg/mL)	10 μM, 20 μM, 40 μM	Oxidative stress; cytokine concentrations; NF-κB pathway	↑ ECM production; ↓ ECM degradation; ↓ Pro-inflammatory cytokines; ↓ Oxidative stress	[67]
		In vivo	DMM in C57BL/6 mice	20 mg/kg/day by gastric perfusion for 8 weeks			
	Galangin	In vitro	Human OA primary chondrocytes	0.01 μM, 0.1 μM, 1 μM, 10 μM, 100 μM	ERK pathway; Oxidative stress	↑ ECM production; ↓ ECM degradation; ↓ Oxidative stress;	[68]
		In vivo	DMM in male SD rats	20 mg/kg, 40 mg/kg, 60 mg/kg, twice a week for 4 weeks			
	Hyperoside	In vitro	murine C57BL/6 chondrocytes exposed to IL-1β (10 ng/mL)	10 μM, 20 μM, 40 μM	Oxidative stress; Apoptosis; NF-κB pathway; ERK pathway; JNK pathway; P38 pathway;	↓ ECM degradation; ↓ Apoptosis	[69]
		In vivo	DMM in C57BL/6 mice	20 mg/kg/day Injected intraperitoneally for 4 or 8 weeks			
	Icaarin	In vitro	SW1353 cell line exposed to IL-1β (5 ng/mL, 10 ng/mL)	5 μM, 10 μM, 20 μM, 40 μM, 80 μM, 100 μM	mTOR pathway; Autophagy;	↑ ECM production; ↓ ECM degradation; ↑ Autophagy	[70]
		In vitro	CHON-001 cell line and ATDC5 cell line exposed to IL-1β (1 ng/mL, 5 ng/mL, 10 ng/mL, 20 ng/mL)	10 μM, 20 μM, 30 μM	cytokine concentrations; apoptosis;	↓ Pro-inflammatory cytokines; ↑ Proliferation; ↓ Apoptosis	[71]
		In vitro	ADTC5 cell line exposed to TNF-α (20 ng/mL)	0.1 μM, 1 μM, 10 μM	NF-κB pathway;	↓ ECM degradation;	[72]
		In vivo	Articular cartilage defect model in C57BL/6 mice	1 μM implanted in alginate-gelfoam complexes			
		In vitro	SW1353 cell line exposed to IL-1β (10 ng/mL)	1 μM, 5 μM, 10 μM, 20 μM, 40 μM, 80 μM, 100 μM	Apoptosis; mTOR pathway; Autophagy;	↓ Apoptosis; ↓ Cartilage degeneration	[73]
		In vivo	ACLT in male SD rats	20 mg/kg/day; 40 mg/kg/day; 80 mg/kg/day; intraperitoneal injection for 4 weeks			
		In vivo	Articular cartilage defect model in New Zealand white rabbits	0.94 g/kg/day; intra-articular injection	-	↑ Articula cartilage repair; ↑ ECM production; ↓ ECM degradation;	[74]
	Quercetin	In vitro	Primary rat chondrocyte exposed to IL-1β (10 ng/mL)	4 μM, 8 μM	mTOR pathway; Autophagy;	↑ ECM production; ↓ ECM degradation; ↑ Autophagy; ↓ Apoptosis;	[75]
		In vivo	ACLT in male SD rats	6 μM or 8 μM (100 μL) in articular cavity once a week for 6 weeks			
		In vivo	ACLT in SD rats	50 mg/kg/day; 100 mg/kg/day; 200 mg/kg/day	mTOR pathway;	↓ Pro-inflammatory cytokines; ↓ ECM degradation;	[76]
		In vivo	MIA-induced OA in SD rats	25 mg/kg/day; 50 mg/kg/day; 100 mg/kg/day; Intragastric administration	-	↓ Pro-inflammatory cytokines; ↓ ECM degradation; ↑ ECM production; ↓ subchondral bone damage; ↓ bone loss	[77]
	Rutin	In vitro	Primary human chondrocyte exposed to advanced glycation end products (AGEs, 50 μg/mL)	10 μM, 20 μM, 40 μM	Oxidative stress; Apoptosis; NF-κB pathway; ERK pathway; JNK pathway; P38 pathway;	↑ ECM production; ↓ ECM degradation; ↓ Pro-inflammatory cytokines	[78]
		In vivo	DMM in C57BL/6 mice	40 mg/kg/day; via oral route for 8 weeks			
Isoflavones	Biochanin A (BCA)	In vivo	DMM in C57BL/6 mice	20 mg/kg; 40 mg/kg; Intragastrically once a week for 8 weeks	Nrf-2 pathway;	↓ Apoptosis; ↓ Oxidative stress; ↑ ECM production; ↓ ECM degradation;	[79]
	Calycosin	In vitro	Human primary chondrocytes exposed to IL-1β (10 ng/mL)	1 μM, 5 μM, 10 μM, 20 μM,	mTOR pathway; cytokine concentrations; Apoptosis;	↓ Pro-inflammatory cytokines; ↓ Apoptosis	[80]
		In vitro	Primary mouse chondrocyte exposed to IL-1β (10 ng/mL)	12.5 μM, 50 μM, 100 μM, 200 μM, 400 μM	NF-κB pathway; cytokine concentrations; Oxidative stress; Apoptosis;	↑ ECM production; ↓ ECM degradation; ↓ Pro-inflammatory cytokines	[81]
		In vivo	DMM in C57BL/6 mice	40 mg/kg/day intraperitoneally for 8 weeks			
	Caviunin glycoside (CAFG)	In vitro	Primary rat chondrocyte exposed to IL-1β (10 ng/mL)	100 pM, 1 nM, 10 nM, 100 nM, 1 μM	Apoptosis; Oxidative stress;	↓ ECM degradation; ↓ Pro-inflammatory cytokines; ↓ Apoptosis; ↓ ROS generation;	[82]
		In vivo	MIA-induced OA in SD rats	250 mg/kg/day; 500 mg/kg/day; oral gavage for 28 days			
	S-Equol	In vitro	rat primary chondrocytes exposed to sodium nitroprusside (SNP; 0.8 mM)	1 μM, 3 μM, 10 μM, 30 μM, 50 μM,	Apoptosis; Oxidative stress;	↑ Proliferation; ↓ Apoptosis; Attenuated ↓ ECM degradation; ↓ NO and H_2_O_2_ production	[83]
	Glabridin	In vitro	Human OA chondrocytes	0.01 μM, 0.1 μM, 1 μM, 5 μM	Oxidative stress; Apoptosis; mTOR pathway; Autophagy; Cytokine concentrations;	↑ ECM production; ↓ ECM degradation; ↓ Pro-inflammatory cytokines; ↓ ROS generation; ↑ Autophagy;	[84]
		In vivo	ACLT in male SD rats	1 mg/kg, 5 mg/kg, 10 mg/kg twice a week for 4 or 8 weeks			
	Neobavaisoflavone (NBIF)	In vitro	Rat chondrocytes exposed to IL-1β (20 ng/mL)	0.25 µM, 0.5 µM, 1 µM, 5 µM, 25 µM, 50 µM, 100 µM, 200 µM	Apoptosis; Cytokine concentrations; Oxidative stress; NF-κB pathway;	↓ Apoptosis; ↓ Pro-inflammatory cytokines; ↓ ROS generation;	[85]
		In vivo	DMM in rats	30 mg/kg/day oral gavage for 7 days			
	Ononin	In vitro	rat primary chondrocytes exposed to IL-1β (10 ng/mL)	1 nM, 10 nM, 100 nM, 1 μM, 10 μM, 100 μM	cytokine concentrations; Oxidative stress; Apoptosis; NF-κB pathway; ERK pathway; JNK pathway; P38 pathway;	↑ ECM production; ↓ ECM degradation; ↓ Pro-inflammatory cytokines	[86]

## 4. The Perspectives on the Use of Flavonoids in Osteoarthritis of Aged Population

Considering all the selected works studying the flavonoids’ activity, only a few aspects have been evaluated. All the studies almost exclusively took into consideration the inhibition of the degradation of the ECM of the cartilage, evaluating the expression of the enzymes responsible for its degradation (e.g., MMP-13), as well as the reduction in the inflammatory state (e.g., IL-1β, IL-6, TNF-α, etc.), the activation of the NF-κB pathway, and their activities that reduce the oxidative state, also through the observation of Nrf-2 activation. In terms of the groups of flavonols and isoflavones, the researchers evaluated the ability of these molecules to induce the production of the cartilaginous extracellular matrix, as well as the inhibition of inflammation-induced chondrocyte apoptosis. Finally, very few manuscripts took into consideration other aspects related to OA, such as the induction of autophagy to avoid apoptosis, reduction in senescence, fibrosis reduction, as well as chondrocyte hypertrophy and osteoclastogenesis, which degrades the subchondral bone and its vascularization. Lastly, only three molecules were investigated for their nutrigenomic effect. Although many works highlighted how many phytochemicals have an anti-osteoarthritic activity, few molecules have been investigated in all aspects related to the disease. For example, since inflammation is able to alter DNA methylation of pro-inflammatory genes, increasing their expression in several biological systems [87,88,89], and considering the nutrigenomics effects of dietary compounds on DNA methylation even in pathologies with inflammatory etiologies [90,91], further nutrigenomic in vitro studies and in vivo preclinical investigations, with the support of in silico studies, will be of great help in the future use of the flavonoids as treatment or co-treatment of OA. The main limit to their use as anti-arthritic drugs (see Outstanding Issues) is due to the lack of specific studies on the real mechanisms of action on cartilage in patients with OA.

Despite the continuous increase in knowledge on their mechanisms of action, obtained from preclinical in vitro and in vivo, as well as in silico, studies, currently there are no flavonoids marketed as supplements for OA treatment. In this regard, we think that to obtain mechanistic information, it will be necessary to further investigate the nutrigenomic effects of almost all of these phytochemicals. Only with these data will it be possible to overlap pharmacological and nutraceutical properties to have additive and enhancing outcomes of OA therapy. Clearly, in addition to having the purpose of mitigating an ongoing pathology by reducing the inflammatory and oxidative processes, and consequently attenuating the destruction of cartilage tissue, it could be useful for the compounds to also have an effect on the deposition of new matrix and anti-senescence activity, and on the inhibition of apoptosis, allowing not only a slowdown but possibly a regression of the pathology, with a recovery from disability. Therefore, regarding in vivo studies, for some of these compounds (such as C3G, baicalein, wogonoside) preclinical studies have almost been completed, confirming their potential role in OA of the aged population, while more testing is needed for others. Further preclinical data are needed to compensate for the deficiencies in understanding the mechanisms of action of these compounds before a clinical trial can be considered as part of a real therapy plan.

## 5. Discussion

Flavonoids are the most abundant polyphenols with health-beneficial activities. Natural flavonoids have been explored in this systematic review of therapeutic strategies to prevent the development and progression of OA. Flavonoids have unequivocally demonstrated their protective actions aimed at hindering pathological cartilage degradation, highlighting specific effects primarily on chondrocytes and cartilage, but also on synoviocytes and subchondral bone, where they interfere with the same pathways. These aspects are of particular importance in the elderly and obese populations, which physiologically develop “inflammaging”, a low grade of systemic inflammation, which is a possible cause of the development and progression of OA. For these reasons, age and overweight represent the strongest risk factors for OA, since they lead to a reduction in the regenerative capacity of chondrocytes, as well as in the maintenance of cartilage homeostasis, where the accumulation of risk factors perturb this equilibrium [91]. Initially, chondrocytes were the only cell type considered in OA etiopathogenesis, and as cartilage is not vascularized, local or systemic inflammation was not taken into account. Successively, new evidence has led to the insight that other cell types and tissues are involved in OA pathogenesis and progression, such as synoviocytes, immune system cells, and subchondral bone. In fact, inflammatory mediators induce cartilage degradation and the loss of chondrocytes, leading to OA’s initiation and development. In addition, changes in ECM contents, such as the Col10A1 produced by hypertrophic chondrocytes, induced by biochemical and biomechanical signals (inflammation, oxidative stress, and joint overload), can act as promoters of OA [92]. In the elderly population, the basal level of inflammation typically experiences an age-related elevation, resulting in detrimental effects. The inflammaging is a consequence of various intrinsic and extrinsic stimuli. It is characterized by an increased production of pro-inflammatory molecules, IL-6 in particular, and a decreased ability to regulate the immune response. Inflammaging plays a significant role in the development and progression of osteoarthritis, as chronic inflammation in the joint can lead to cartilage breakdown, joint pain, and functional impairment. Over time, this progressive elevation in inflammatory status serves as a significant risk factor for multiple diseases [93]. The heterogeneity observed in the process of biological aging arises from a combination of genetic factors, environmental influences (such as pollution and habitat), and lifestyle habits (including nutrition, exercise, and light exposure). These factors significantly influence the manifestation of aging phenomena in the elderly population, as well as in the obese population. Notably, prolonged periods of excessive caloric intake (overnutrition) or inadequate caloric intake (malnutrition) can trigger an upregulation of the inflammatory response, thereby leading to a chronic inflammatory state, particularly in older individuals [94]. It is known that vitamin D plays a role in cartilage and bone metabolism, and it has been hypothesized that low levels may increase OA risk. Furthermore, the actions of vitamin D in the regulation of the immune system, reducing pro-inflammation mediators and inducing anti-inflammatories, could mitigate OA progression. The VIDEO study, a randomized double-blind trial, compared a group of patients that received a placebo with a group of patients that assumed vitamin D for two years. The results showed stable synovitis in patients with vitamin D treatment, while it increased in the placebo group [95].

Numerous studies have highlighted that the consumption of high-fat meals, both in healthy individuals and the elderly, can elevate the levels of serum lipopolysaccharides (LPSs) produced by gut microbiota. Subsequently, this LPS stimulates the activation of leukocytes in the innate immune system, leading to the production of pro-inflammatory molecules such as TNF, IL-1β, and IL-6, thereby initiating an inflammatory state [94]. Clearly, the relationship between inflammaging and osteoarthritis is complex and multifaceted, but this association is due to elevated systemic and local inflammations, which are naturally increased in the elder population but could be accelerated by several other factors, that promote cartilage degradation and OA pathogenesis and progression [95]. Pro-inflammatory cytokines such as TNFα, IL-1β, and IL-6 accelerate the aging process of mesenchymal stem cells, impairing their ability to differentiate into other cells and subsequently alter osteochondral homeostasis in the elderly population, leading to osteoarthritis. Moreover, these inflammatory factors aggravate osteoarthritis by activating specific pathways and stimulating the release of inflammatory mediators with the final problematic consequence of OA symptoms being aggravated [96,97].

It is known that oxidative stress induces the acceleration of cellular aging that determines a reduction in functionality. ROS production accelerates senescence in chondrocytes, resulting in terminal differentiation and apoptosis. Repeated exposures to ROS affect the shortening of telomers, which represents the best indicator of senescence in the cells. Telomere attrition down to a critical length leads to cell cycle arrest and activation of apoptosis. DNA damage directly resulting from oxidative damage is responsible for telomere attrition. This is due to the fact that oxidant insults preferentially attack GGG sequences, in which the telomeric sequences are rich. Blocking of replication due to altered DNA sequences, such as with the presence of unrepaired nucleotides such as oxidized bases, abasic sites, or nucleotide gaps, is represented to be one of the reasons for oxidative stress-induced telomere loss. Dysfunctional telomeres initiate cell cycle arrest and, because modified telomer sequences are recognized as DNA strand breaks, the cells go into apoptosis. Then, short telomeres are more susceptible to varying stressors, such as oxidative stress [98].

**Figure 6:**
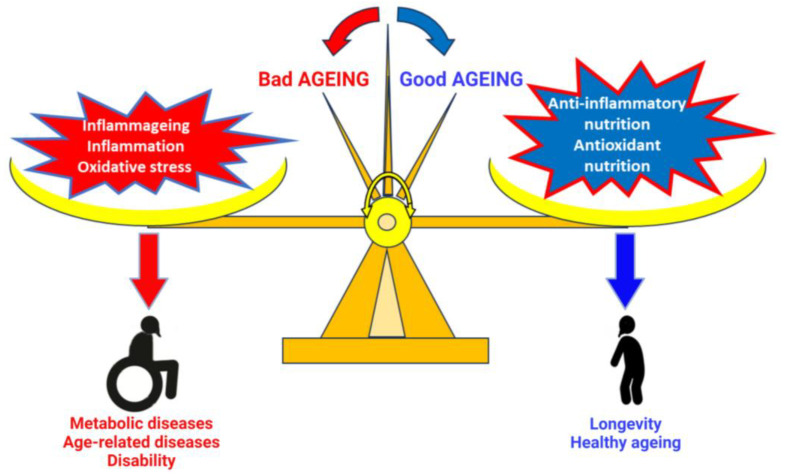
Schematic drawing representing the balance between good and bad aging that could be helped by phytochemical compounds with antioxidant and anti-inflammatory nutrigenomic activities (created with biorender.com).

Life expectancy has increased in recent decades, while life expectancy in good health has not had the same increase, as indicated by the significant presence of pathologies in old age. Recent evidence has shown how inflammaging has contributed to the increased risk of developing chronic diseases. Furthermore, the effects of obesity in terms of inflammaging can be comparable to those observed in elderly individuals, suggesting that at a molecular level, elderly and obese subjects may exhibit similar characteristics. Moreover, a few studies have reported how an increase in circulating anti-inflammatory cytokines could counterbalance inflammaging, while these findings are partially contradicted by other articles, where observations are made of an age-associated decline in circulating levels of IL-10. By targeting the underlying inflammatory processes associated with inflammaging, it may be possible to alleviate symptoms, slow down disease progression, and improve the quality of life for individuals living with osteoarthritis. Flavonoids are known to contrast with inflammation and oxidative stress, and, consequently, they are able to reduce the low-grade chronic inflammation typical of inflammaging in the aged population, contrasting, then, with all age-related pathologies such as OA. Recent experimental evidence has brought to light the role of some flavonoids in modulating aging. In fact, with their protective, antioxidant, and anti-inflammatory properties, these molecules have been described in several reviews as therapeutic agents, acting via the inhibition of NF-kB and phosphorylation of MAPKs, but descriptions have also been offered of their epigenetically related actions such as DNA methylation, histone modification, and noncoding of RNA [99,100]. By performing nutrigenomic interventions, the unique approach that is potentially able to influence OA molecular pathways, it will be possible to moderate the OA composite’s pathological features, and we will gain ameliorations in the initiation, progression, and prognosis of this systemic disease (see Figure 6) [101,102].

## 6. Conclusions

Although the efficacy of the discussed flavonoids has been demonstrated, the therapeutic efficacy of using a single molecule for managing OA in the elderly population may be limited due to the complexity of the pathology. A combination of multiple molecules or their symbiotic use with other drugs may be a more effective approach. Unfortunately, the information on single molecules is revealed in a fragmentary manner and, as already discussed, not all mechanisms have been explored. Therefore, the use of a strategy involving multi-drug therapies is rather limited. Although we have demonstrated the multifaceted pharmacological activities of flavonoids, there is still a need for numerous clinical studies to integrate this currently preclinical information. Undoubtedly, significant progress has been made in studying the therapeutic role of flavonoids in OA. Nevertheless, the metabolism of flavonoids and all the nutrigenomic aspects of these molecules are yet to be fully understood. Future investigations on osteoarthritis (OA) should explore the pathology from multiple perspectives. This would enable a comprehensive understanding of the activity that takes place at both the local level in articular cartilage and subchondral bone, as well as at the systemic level. This approach is necessary since the molecule under study might also interact positively or negatively with other physiological aspects or co-occurring pathologies. It is a well-established fact that osteoarthritis in the elderly frequently co-occurs with other pathologies, regardless of whether they are inflammatory in nature or not.

Recently, treatment strategies for OA have been developed that target senescent cells and the paracrine and autocrine secretions of the senescence-associated secretory phenotype [102]. Our labs have already ascertained the nutrigenomic effects of some molecules contained in plants, highlighting their effects on inflammation and autophagy, and their power to counteract epi-mutagenic actions [90,103,104,105,106], for example, arsenic-induced actions [106]. We therefore believe that the nutrigenomic approach represents the most modern frontier for understanding the action of flavonoids in OA.

## Data Availability

The data that support the findings of this review are available from the corresponding author upon reasonable request.

## Abbreviations

A disintegrin and metalloproteinase with thrombospondin motifs—ADAMTS; Adenomatous Polyposis Coli—APC; aggrecan—ACAN; Ak strain-transforming protein—AKT; AMP-activated protein kinase—AMPK; angiotensin II—ANG-II; anterior cruciate ligament transection—ACLT; apoptosis-associated speck-like protein—ASP; apoptosis-inducing factor mitochondria-associated 1—AIFM1; B-cell leukemia/lymphoma 2 protein—BCL-2; B-cell lymphoma extra-large protein—BCL-XL; BCL-2-associated X protein—BAX; Biochanin A—BCA; BMP receptor—BMPR; Bone Morphogenic Proteins—BMPs; carboxy-terminal telopeptides of type II collagen—CTX-II; cartilage oligomeric matrix protein—COMP; caviunin glycoside—CAFG; c-Jun N-terminal kinase—JNK; collagen—Col; collagen-induced arthritis—CIA; Core-Binding Factor-1—CBF-1; cyanidin-3-glucoside chloride—C3G; cyclooxygenase-2—COX-2; Cytoskeleton Regulator RNA—CYTOR; diacylglycerol—DAG; Dikkopf—Dkk; diosmetin—DIOS; Epigallocatechin-3-O-gallate—EGCG; extracellular matrix—ECM; extracellular signal-regulated kinase—ERK; fatty acid-binding protein-4—FABP-4; fibroblast-like synovial cells—FLSs; forkhead box protein O-1—FoxO1; galangin—Gal; glutathione—GSH; Glycogen Synthase Kinase-3β—GSK-3β; Hedgehog—Hh; Heme Oxygenase-1—HO-1; HOX transcript antisense RNA—HOTAIR; human umbilical vein endothelial cells—HUVECs; hypoxia-inducible factor—HIF; icariin—ICA; inducible nitric oxide synthase—iNOS; inositol triphosphate—IP3; interferon—IFN; interleukin—IL; isoliquiritigenin—ISL; IκB kinase—IKK; licochalcone A—Lico A; lipids peroxide oxidation—LPO; lipopolysaccharide—LPS; lipoprotein lipase—LPL; Low-Density Lipoprotein 5/6—LRP 5/6; lymphoid enhancer binding factor—LEF; malondialdehyde—MDA; mammalian target of rapamycin—mTOR; matrix metalloproteinases—MMPs; micro-computed tomography—μCT; microtubule-associated protein 1A/1B light chain 3—LC3; mitogen-activated protein kinase—MAPK; mono-iodoacetic injection—MIA; myeloid differentiation factor 2—MD-2; naringenin—NAR; neobavaisoflavone—NBIF; Notch intracellular domain—NICD; nuclear factor erythroid 2-related factor-2—Nrf-2; nuclear factor of kappa light polypeptide gene enhancer in B-cells inhibitor—IκB; nucleotide-binding domain, leucine-rich–containing family, pyrin domain–containing-3—NLRP-3; nucleus pulposus cells—NPCs; osteoarthritis—OA; Osteoarthritis Research Society International—OARSI; ovariectomy—OVX; oxidative stress—OS; Patched-1—PTC-1; pelargonidin—PG; phosphatase and tensin homolog—PTEN; phosphatidyl inositol diphosphate—PIP2; phosphatidyl inositol triphosphate—PIP3; phosphatidylinositol 3-kinase—PI3K; phospholipase C—PLC; procyanidin B2—PCB2; proline- and arginine-rich end leucine-rich repeat protein—PRELP; prostaglandin E2—PGE-2; prostaglandin-endoperoxide synthase-2—PTGS-2; protein kinase C—PKC; Quercetin—Que; RANK ligand—RANKL; Ras Homolog Enriched in Brain 1—RHEB; Reactive Oxygen-containing Species—ROS; receptor activator of NF-κB—RANK; regulatory T—T-reg; rhoifolin—ROF; runt-related transcription factor 2—RUNX-2; safflower yellow—SY; Secreted Frizzled-Related Protein—sFRP; senescence-associated secretory phenotype—SASP; Sirtuin-6—Sirt-6; SMA- and MAD-Related Protein—Smad; Smad ubiquitination regulatory factor 1—SMURF-1; smoothened—SMO; sodium nitroprusside—SNP; SRY-Box Transcription Factor 9—SOX-9; superoxide dismutase—SOD; surgical destabilization of medial meniscus—DMM; synovial membrane-derived mesenchymal stem cells—SMMSCs; T-cell factor—TCF; Theaflavin-3,3′-Digallate—TFDG; toll-like receptor—TLR; Tuberous Sclerosis Complex 2—TSC2; Tumor Growth Factor-β—TGF-β; tumor necrosis factor receptor-associated factor—TRAF; tumor necrosis factor-α—TNF-α; Unc-51-like autophagy-activating kinase—ULK1; vascular endothelial growth factor A—VEGFA; wingless-type MMTV integration site family member—Wnt; xanthohumol—XN.
